# Elimination of human rabies in Goa, India through an integrated One Health approach

**DOI:** 10.1038/s41467-022-30371-y

**Published:** 2022-05-19

**Authors:** A. D. Gibson, G. Yale, J. Corfmat, M. Appupillai, C. M. Gigante, M. Lopes, U. Betodkar, N. C. Costa, K. A. Fernandes, P. Mathapati, P. M. Suryawanshi, N. Otter, G. Thomas, P. Ohal, I. Airikkala-Otter, F. Lohr, C. E. Rupprecht, A. King, D. Sutton, I. Deuzeman, Y. Li, R. M. Wallace, R. S. Mani, G. Gongal, I. G. Handel, M. Bronsvoort, V. Naik, S. Desai, S. Mazeri, L. Gamble, R. J. Mellanby

**Affiliations:** 1 Mission Rabies, Cranborne, Dorset, United Kingdom; 2 The Roslin Institute and The Royal (Dick) School of Veterinary Studies, The University of Edinburgh, Easter Bush Veterinary Centre, Roslin, Midlothian, United Kingdom; 3 Mission Rabies, Tonca, Panjim, Goa, India; 4 Poxvirus and Rabies Branch, Centers for Disease Control and Prevention, Atlanta, GA USA; 5 Department of Animal Husbandry & Veterinary Services, Government of Goa, Panaji, India; 6 Directorate of Health Services, Government of Goa, Panaji, India; 7 Worldwide Veterinary Service India, Ooty, Tamil Nadu India; 8 LYSSA LLC, Atlanta, Georgia United States; 9 Merck Animal Health, Madison, NJ USA; 10 MSD Animal Health, Walton Manor, Walton, Milton Keynes, MK7 7AJ United Kingdom; 11 Department of Neurovirology, WHO Collaborating Centre for Reference and Research in Rabies, National Institute of Mental Health and Neurosciences, Bengaluru, India; 12 WHO Regional Office for South East Asia, New Delhi, India

**Keywords:** Viral genetics, Viral epidemiology, Developing world, Epidemiology

## Abstract

Dog-mediated rabies kills tens of thousands of people each year in India, representing one third of the estimated global rabies burden. Whilst the World Health Organization (WHO), World Organization for Animal Health (OIE) and the Food and Agriculture Organization of the United Nations (FAO) have set a target for global dog-mediated human rabies elimination by 2030, examples of large-scale dog vaccination programs demonstrating elimination remain limited in Africa and Asia. We describe the development of a data-driven rabies elimination program from 2013 to 2019 in Goa State, India, culminating in human rabies elimination and a 92% reduction in monthly canine rabies cases. Smartphone technology enabled systematic spatial direction of remote teams to vaccinate over 95,000 dogs at 70% vaccination coverage, and rabies education teams to reach 150,000 children annually. An estimated 2249 disability-adjusted life years (DALYs) were averted over the program period at 526 USD per DALY, making the intervention ‘very cost-effective’ by WHO definitions. This One Health program demonstrates that human rabies elimination is achievable at the state level in India.

## Abstract (web summary)

Dog vaccination is an effective rabies prevention measure, but widespread vaccination campaigns are challenging in settings like India with large free-roaming dog populations. Here, the authors describe a One Health campaign in Goa state which led to a large reduction of cases in dogs and elimination in humans.

## Introduction

Rabies is a devastating and societally important zoonotic disease, which is transmitted principally to humans through the bite of infected dogs. This acute, progressive viral encephalitis has the highest case fatality of any infectious disease and kills tens of thousands of people annually, with children and impoverished communities being affected disproportionately^1,2^.

India is estimated to suffer the greatest rabies burden of any country, both in terms of annual human deaths and disability-adjusted life years (DALYs)^1^. Although the timely delivery of human post-exposure prophylaxis (PEP) prevents death from rabies, focusing on the post-bite treatment of people (a dead-end host) has no impact on the incidence of rabies in the canine reservoir population, leaving other members of the community vulnerable to acquiring the disease^3^. The effectiveness of mass dog vaccination in eliminating rabies from the reservoir animal population, and thereby preventing viral transmission to humans, has been known for over a century^4^, enabling dog-mediated rabies to be eliminated in numerous countries^5–7^. Modern rabies management highlights the importance of achieving zoonotic disease prevention and control through consideration of human, animal, and environmental components in a One Health approach^8^.

In most endemic settings without vaccination, the reproductive number of rabies in dogs is below two under natural conditions, falling below one where over 40% of the dog population is vaccinated^9^. To account for population turnover, annual vaccination of over 70% of dogs has been shown to successfully eliminate viral perpetuation, making canine rabies an ideal candidate for worldwide elimination^9,10^. Due to the particular ecology of dogs in India, where millions of dogs are free-roaming and hard-to-reach^11^, rabies is considered very challenging to eliminate, as is reflected in the complete paucity of examples of rabies elimination in any Indian state^12^. The reasons for this failure are invariably multifactorial, but achieving a step-change in the political prioritization of rabies control and surmounting the logistical challenges in reaching vaccination coverages sufficient to control the disease is critical to the quest for canine rabies elimination at the state level^11–13^.

Here, we report how these challenges were overcome in Goa, India through a collaboration between local government, non-governmental organizations, and academic partners, culminating in the elimination of human rabies, for the first time, at the state level in India. The One Health program consisted of three core areas of activity: dog vaccination; rabies education; and intensified human and animal rabies surveillance.

## Results

### Dog vaccination

**Fig. 1:**
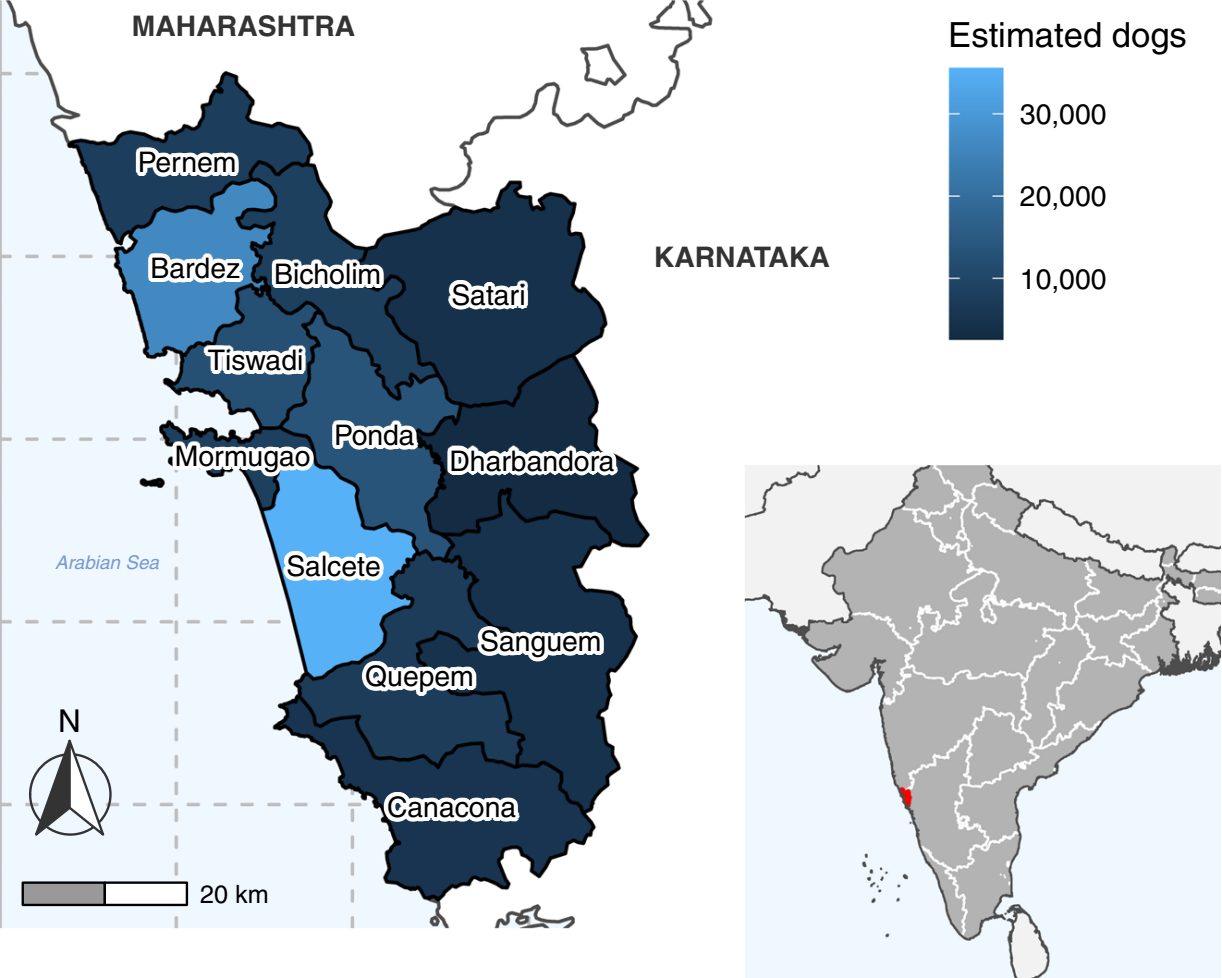
Map of Goa state estimated dog population by taluka. Choropleth map of Goa state showing taluka boundaries and colored by the estimated dog population. Dog population estimates were calculated from mean vaccination coverage and mean number of dogs vaccinated per taluka during vaccination cycles in which comprehensive post-vaccination surveys took place (Supplementary Methods, Supplementary Fig. 7). Inset map shows the state borders of India (white lines) and the location of Goa state (red). India state and Goa taluka boundaries were sourced from https://gadm.org.

The central goal of this One Health program was to eliminate human rabies deaths by reducing rabies incidence in the canine reservoir through mass dog vaccination. This was achieved with mobile dog vaccination teams aiming for high coverage in both the free-roaming and owned confined dog populations throughout the state. Remote vaccination teams were spatially directed through assigned polygons displayed on a smartphone app, enabling managers to deliver vaccination resources to a specific geographic area at the sub-village scale^14,15^. The GPS and details of each dog vaccination were recorded offline in the app and subsequently shared with project managers through an administrator website. Vaccination teams rotated through the administrative regions of Goa (talukas) (Fig. 1), re-starting the state campaign cycle on an approximately annual basis (Supplementary Figs. 2–4). A combination of door-to-door (DD) and capture-vaccinate-release (CVR) methods were used to access dogs for parenteral vaccination. DD vaccination involved teams walking house-to-house offering owners an opportunity to have their dog vaccinated, whilst CVR consisted of teams using nets to catch and vaccinate dogs that could not be restrained manually.

**Fig. 2:**
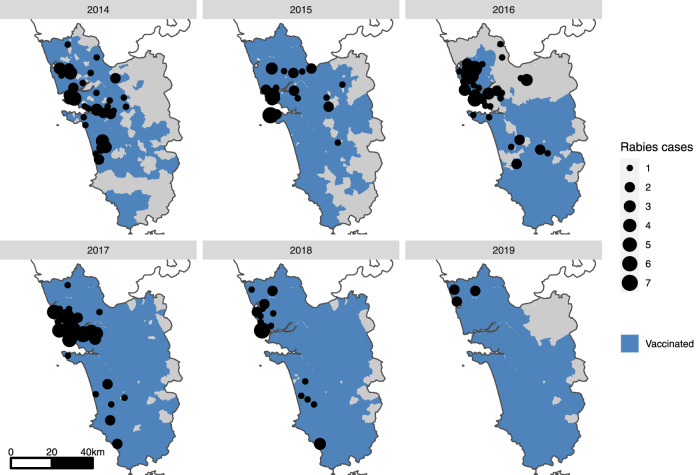
Year-wise maps of Goa state showing canine rabies cases and the extent of dog vaccination. Maps of Goa state (gray shading) showing the villages/municipalities in which vaccination took place each year (blue shading) and positive canine rabies cases by village (black circles). A small-scale pilot dog vaccination campaign was conducted in 2013 (Supplementary Fig. 3), however, no location-specific canine rabies surveillance data were available at this time.

The annual vaccination output increased, both in terms of geographic extent and a total number of dog vaccinations, through program refinement from 2013 to 2017 (Fig. 2). Intensive state-wide vaccination was achieved for the first time in 2017, vaccinating 97,277 dogs in an estimated total population of 137,353 dogs. This output was sustained through 2018 and 2019 (Fig. 2). A total of 426,119 rabies vaccine doses were administered to dogs between 2013 and 2019 (Fig. 2, Supplementary Fig. 3, Supplementary Table 1).

Vaccination methods were evaluated using post-vaccination dog-sight surveys to estimate coverage. A total of 3188 post-vaccination surveys were conducted during the period of study, recording 280,859 dog sightings. Final vaccination coverage was estimated from the last survey of each area, resulting in the omission of 793 surveys from the final analysis. The mean vaccination coverage in the 2016 campaign was 71.8% in all sighted dogs and 60.1% in roaming dogs, however, some areas of the state were not vaccinated. In 2017 intensive methods were applied state-wide achieving an estimated coverage of 71.7% in all dogs sighted and 53.1% in the roaming population (Supplementary Fig. 5).

Both ownership and confinement data were available for over 90% of dog vaccination records (*n* = 384,149), of which 52% (*n* = 199,887) were owned dogs and 48% (*n* = 184,262) were unowned dogs. Unowned dogs were inherently always roaming, while owned dogs were either always roaming (*n* = 35,823, 17.9%), allowed to roam for some of the time (*n* = 100,618, 50.3%) or always confined (never roaming) (*n* = 63,446, 31.7%). Consequently, most dogs vaccinated (83.5%) were among the roaming dog population for some or all of the time (Supplementary Fig. 4). The proportion of dogs vaccinated that were owned differed significantly between urban and rural settings. Of 213,467 dogs vaccinated in urban areas, 45.1% (CI: 44.9–45.4) were recorded as owned, as compared to 60.6% (CI: 60.4– 60.9) of 170,682 dogs vaccinated in rural settings (test of equal proportions *p* < 0.001).

Operational efficiency was improved through iterative refinement of vaccination methods. The initial approach, focusing on net-catching of unowned dogs in 2013, was adjusted to include the vaccination of owned dogs from October 2015. Smaller two-person DD vaccination teams were introduced in 2018 to more efficiently vaccinate dogs that could be restrained by hand, which was the case for 64.2% of all dogs vaccinated (restraint data available from 2018). Nevertheless, specialist equipment was still needed to access a considerable proportion of dogs, with 16.3% of owned dogs and 56.5% of unowned dogs being restrained by a net. The mean number of active vaccination teams per day increased from 2.5 (95% CI: 2.4–2.7) CVR teams in 2016 to a total of 7.7 (CI 7.4–8.0) (4.4 CVR teams and 3.3 DD teams) in 2019. Dog vaccination program-specific costs were available for 2018 and 2019, producing a mean cost per dog vaccinated of 3.45 USD (Supplementary Table 5).

### Rabies education

The second pillar of the program was an education initiative whose primary focus was on teaching school children about rabies, how to avoid dog bites and what to do if bitten. The program also emphasized the importance and social value of ensuring as many dogs as possible were vaccinated against rabies each year. In total, school-based rabies education classes were delivered to 694,271 school children and 31,251 teachers between 2014 and 2019 (Supplementary Table 1). The scale of the school education program increased from 2014, plateauing from 2017 onwards with the delivery of educational lessons to ~170,000 children per year across 1400 schools in Goa. Activities to distribute rabies educational messages throughout communities intensified with a similar timeframe which resulted in the delivery of rabies lessons to 155,079 people through community groups, local authorities, and public events (Supplementary Table 1).

### Dog rabies surveillance

The third pillar of the program was rabies surveillance within the dog population. Enhanced canine rabies surveillance was driven by improving the reporting of suspected rabid dogs from across public and private sectors. This was centrally managed through a widely publicized Rabies Hotline phone service launched in 2014.

Details of phone calls to the Rabies Hotline were available from October 2017, totaling 7372 and increasing from an average of 50.2 calls per week in 2018 (95% CI: 42.9–57.5) to 78.9 calls per week in 2019 (CI: 69.9–87.9) (Supplementary Fig. 8, Supplementary Table 2). Mapping the origin of calls showed widespread engagement with the Rabies Hotline throughout the state (Supplementary Fig. 9). The most common reasons for contacting the Rabies Hotline were requests for vaccination of dogs (44.3%), reporting sick or injured dogs (without typical signs of rabies) (32.4%), and dog nuisance (6.7%). Despite increasing total calls, the rate of calls reporting suspect rabid animals reduced from a mean of 6.7 per month in 2017 to 4.8 in 2018 and 4.5 in 2019 (Supplementary Fig. 8).

Reports of suspect rabid animals initiated a veterinary field investigation to examine the animal and interview people involved or exposed. Systems for the management and testing of suspect rabid animals were established in 2014. Samples were initially shipped to the WHO reference laboratory for rabies in Bangalore (NIMHANS) for direct fluorescent antibody (DFA) test diagnosis. A rapid lateral flow assay (LFA) was used as a field-side tool in screening cases^16^, however, LFA results did not inform human PEP decisions. In 2016, the capacity to perform the DFA was established at the Goa Disease Investigation Unit (Department of Animal Husbandry & Veterinary Services) laboratory to provide routine timely rabies diagnosis (Supplementary Methods).

**Fig. 3:**
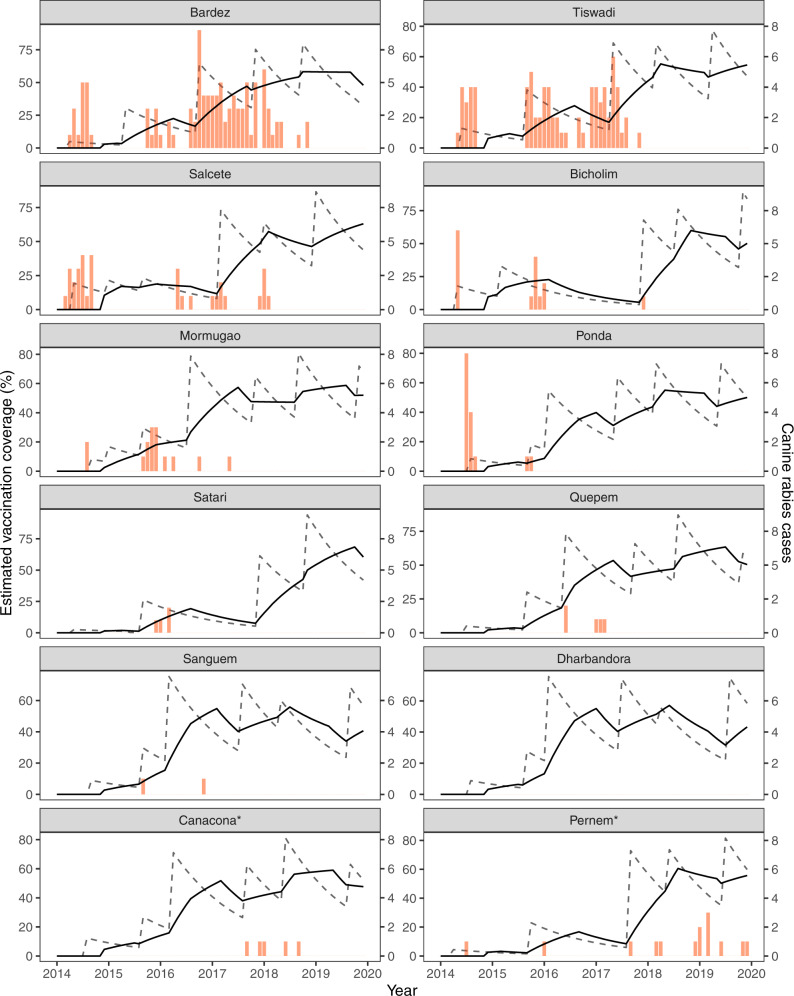
Graph of month-wise estimated vaccination coverage (dotted line), rolling mean 12-month vaccination coverage (solid line), and monthly animal rabies cases (red bars) from 2014 to 2019 for each taluka region of Goa. Asterisk denotes talukas that immediately border areas of high dog density in other states in which rabies remains endemic. Month-wise estimated vaccination coverage was calculated from the number of vaccinations delivered by campaign cycle and total estimated dog population by region, with a month-wise estimate of population turnover.

Seventy-three canine rabies cases were confirmed in the first 6 months of surveillance in 2014, with the highest monthly report of the study period occurring in July 2014 at 20 cases (Supplementary Fig. 10). The mean state-wide occurrence of canine rabies cases decreased from 10.6 cases per month in 2014 to 0.8 in 2019, a decrease of 92% (Supplementary Fig. 10, Supplementary Table 2). The regional distribution of cases also changed significantly across the study period. No canine rabies cases were detected in 11 of Goa’s 12 taluka regions for over a year, from November 2018 until December 2019 (Figs. 2, 3, Supplementary Fig. 11). The continued occurrence of cases in the later stages of the program was confined to the northern region of Goa, bordering unvaccinated dog populations in the neighboring state (Figs. 2, 3).

**Fig. 4:**
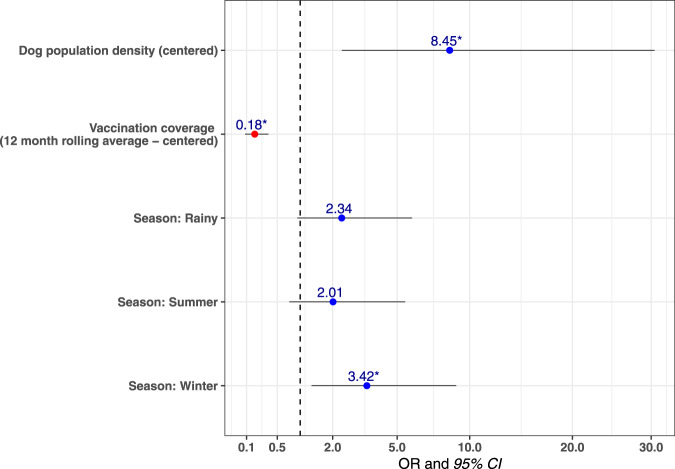
Multivariable mixed-effects logistic regression model for detecting at least one rabies case by taluka in a month. Multivariable mixed-effects logistic regression model predicting a taluka having at least one confirmed dog rabies case in a particular month (*n* = 624). Figure shows the Odds Ratio (points) and 95% confidence intervals (horizontal lines). Asterisks indicate a *p* value < 0.05. The model shows that the odds of a taluka having at least one positive dog rabies case in a particular month increased as roaming dog population density increased. Similarly, as the rolling mean 12-month vaccination coverage increased, the odds of a positive rabies case were reduced. The odds of identifying at least one rabies case during the monsoon season (the reference season in the model) were lower, especially compared to the winter season, which had significantly higher odds. The AUC was calculated as 0.73, indicating that the model was very good at predicting the outcome.

Analysis of the distribution of confirmed canine rabies cases by taluka over time revealed that cases were not homogeneously distributed. Cases predominated in areas of high dog density with a multivariable mixed-effects logistic regression model estimating that the odds of a taluka having at least one confirmed rabies case in a month increased as the taluka’s free-roaming dog population density increased (Fig. 4, Supplementary Table 6). The model also showed that the odds of at least one rabies case occurring decreased as rolling mean 12-month vaccination coverage increased. Two talukas, Pernem and Canacona, neighboring unvaccinated dog populations at the north and south borders of the state did not follow this pattern as canine rabies cases continued to occur in the face of sustained high vaccination coverage (Fig. 3).

**Fig. 5:**
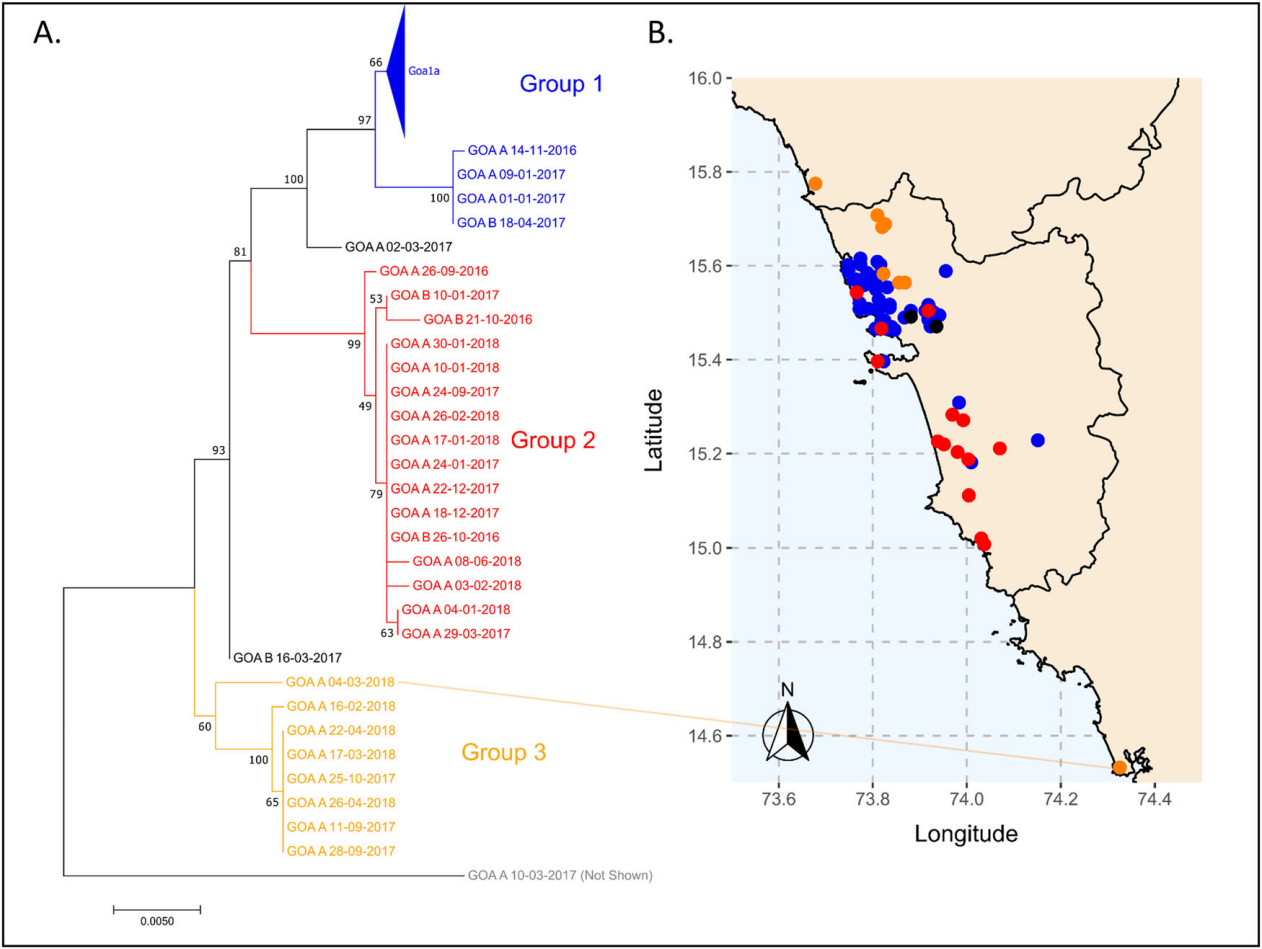
Phylogeographic distribution of canine rabies cases. Phylogenetic analysis of 97 rabies virus glycoprotein gene sequences from Goa, India 2016–2018. The phylogenetic tree was calculated by maximum likelihood (GTR + G + I). Numbers on branch points represent bootstrap support based on 1000 replicates. The colors on the tree correspond to colored points on the Goa map. Sequences in Goa1a are collapsed for viewing convenience; a list of samples in each group is in Supplementary Data 2. Positions on the map are approximate. Some points deviate slightly from their true location to allow for visualization of multiple samples from the same location. The location of sample Goa A 04-03-2018 (Karnataka) is highlighted by a line. The scale bar indicates the number of changes per site.

Rabies virus sequencing was conducted to explore the molecular epidemiology of canine rabies in Goa^17^. Ninety-seven glycoprotein and 80 nucleoprotein gene sequences were generated from samples randomly selected from a bank of rabid dog brain samples spanning 2016 to 2018 (Supplementary Data 2). The sequences formed three major groups based on phylogenetic analysis, average nucleotide identity, and haploid network analysis of the glycoprotein gene: seventy sequences fell into Group 1; sixteen into Group 2; and eight into Group 3 (Fig. 5, Supplementary Fig. 15–17). The high degree of sequence conservation within these groups indicated that they represented co-circulating lineages predominating in discrete geographic regions of Goa state: Group 1 in North Goa; Group 2 in South Goa; and Group 3 in the north border region, with the exception of Goa_A_04-03-2018 (Fig. 5). One sequence, from a rabid dog brought from Karnataka to Goa post-mortem (Goa_A_10-03-2017), was identified as a recently imported case into the region from north India, displaying <97% identity to all other samples (Supplementary Table 7, Supplementary Fig. 16).

**Fig. 6:**
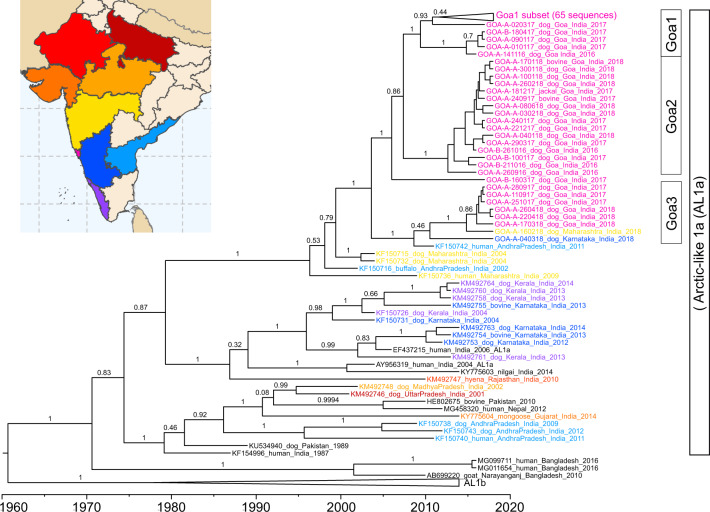
Time-scaled phylogeny for Goa samples and reference samples from other states of India. Time-scaled phylogeny of partial glycoprotein gene sequences (1317 nt) of Goa samples, Karnataka samples, and Maharashtra samples generated in this study with representative reference sequences from India belonging to the Arctic-like 1a rabies virus lineage. Representative samples from the Arctic-like 1b lineages were included as an outgroup. Scale at the bottom indicates year. Sample IDs are colored based on the state of sample collection, according to the coloration on the map. Bars to the right indicate members of Goa1, Goa2, and Goa3 groups. Numbers at the branches indicate posterior support values. AL1b: Arctic-like 1b.

Time-scaled phylogenetic analysis of the glycoprotein gene estimated that Group 1 originated in 2009 (95% CI: 2006.6–2011.7), Group 2 in 2012 (95% CI: 2010.2–2014.6), and Group 3 in 2009 (95% CI: 2005.5–2013.3), with the most recent common ancestor between the groups estimated to be in 2003 (1999.4–2007.3) (Figs. 5, 6). A sub-group of Group 3, excluding sample GOA_A_04-03-18 from Karnataka, was estimated to arise around 2014. Similar estimates (within 2.5 years) were obtained using the complete nucleoprotein gene (Supplemental Fig. 19).

All Goa sequences generated in this study clustered within the Arctic-like 1a rabies virus variant lineage and were most similar to sequences from the neighboring states of Karnataka, Maharashtra, and Andhra Pradesh (Fig. 6). Samples in Groups 1 and 2 clustered separately from sequences generated in other studies. However, Group 3 samples clustered with a rabies virus reference sequence from a human case in Andhra Pradesh, India in 2011 (Fig. 6).

Additional statistical cluster analysis of the spatio-temporal distribution of rabies cases identified one statistically significant cluster centered in North Goa from 2016 to 2017 (Supplementary Figs. 20 and 21). This statistically significant rise in canine rabies, above what would be expected if cases were distributed equally according to the population at risk, occurred where the Group 1 and Group 3 rabies virus lineages coincided. This region was close to the northern border where the Goan dog population interacts with unvaccinated dogs in neighboring districts of Maharashtra (Supplementary Fig. 21).

### Public health impact

**Fig. 7:**
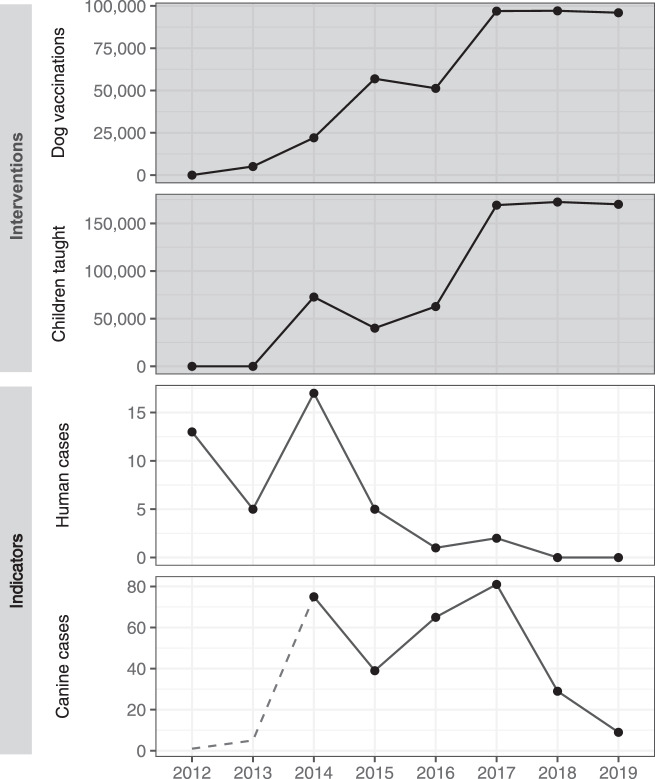
Year-wise graphs of intervention outputs and indicators of rabies control from 2012 - 2019. Graphs show intervention outputs (dark background) of annual total dog vaccinations and children taught about rabies and indicators of rabies control (light background) of annual human rabies deaths and confirmed canine rabies cases. The dotted line in canine cases indicates a period prior to the enhancement of animal rabies surveillance activities. The decrease in canine rabies cases in 2015 is due to the cessation of canine rabies surveillance activities between October 2014 and September 2015.

To assess the human impact of the One Health program, information on dog bites and human rabies deaths was acquired from the Directorate of Health, Government of Goa. Human rabies deaths reduced from 17 in 2014 to zero in 2018 and 2019 (Fig. 7, Supplementary Table 2). This decline in human deaths occurred during a period of increased human rabies surveillance resulting from numerous rabies-focused activities engaging the human medical profession in Goa (Supplementary Methods). The number of reported dog bites increased from 785 per 100,000 people in 2012 to 1430 per 100,000 people in 2019 (Supplementary Fig. 22, Supplementary Table 2).

A previously published model (RabiesEcon^18,19^) was used to estimate the cost-effectiveness of the intervention. Goa-specific programmatic data were used to populate the model, including human and dog population data; annual dog vaccination coverage; program costs; PEP costs; and estimated rates of access to PEP (Supplementary Data 3, Supplementary Table 5). The estimated mean cost per death averted and cost per DALY averted from 2013 to 2019 were 14,866 USD and 526 USD respectively. During this period the program was estimated to result in a total of 2,249 DALYS averted, and 80 deaths averted as compared to no intervention. Over a 10-year projection (2013–2023), the intervention was estimated to prevent 121 human rabies deaths and 3427 DALYS at a mean cost of 567 USD per DALY averted. The model predictions of human rabies deaths, human rabies exposures, and total estimated intervention cost were concordant with empirical values from the program area.

## Discussion

This collaborative One Health program demonstrates the feasibility and cost-effectiveness of human rabies elimination at the state level in India, offering a tangible example of success in the quest to eliminate canine-mediated human rabies deaths by 2030. Despite the terrible toll rabies exerts in India, the inaccessible nature of the huge free-roaming dog population resulted in the modern discourse that, while local elimination of rabies within restricted communities is feasible, extending these approaches to a state-level still presents a near-insurmountable practical and logistical challenge^8,11,20,21^. Such failings resulted in calls to the scientific community to refocus rabies research towards programs that advanced the field implementation and evaluation of rabies elimination strategies^22,23^. The current initiative integrating mass dog vaccination, education, and rabies surveillance, underpinned by innovations in mHealth remote team direction, data capture, and real-time analysis, specifically addressed this challenge and has shown robustly that freedom from dog-mediated human rabies deaths is feasible and within reach.

Dog accessibility is a major barrier to the efficient application of high-coverage state-wide dog vaccination campaigns in India. Whilst central point dog vaccination approaches have achieved high-number, high-coverage outputs in some African settings^10,24^, the high proportion of stray dogs in India limits the likely success of such methods in achieving herd immunity against rabies^11^. Similarly, DD campaigns centered on catching dogs by hand had limited success^25^. The majority of dogs vaccinated in the Goa program were caught by hand, however, achieving adequate vaccination coverage was not possible without specialist equipment and expertise through net-catching. Advanced vaccination techniques are therefore necessary to interrupt rabies virus transmission in such settings. Regular assessment of post-vaccination coverage during the early stages of program development proved to be critical in identifying where the vaccination approach was successful and where improvement was needed^26^. The mean cost of 3.45 USD per dog vaccinated was higher than reported global averages (2.18 USD), but within the range reported by other mass dog vaccination interventions (1.13–15.62 USD)^27,28^. This appears reasonable given the large free-roaming dog population in Goa and reliance on net capture to reach adequate vaccination coverages. The use of oral rabies vaccination (ORV) of dogs in combination with parenteral methods offers the potential to further optimize the current approach to intensively vaccinate otherwise inaccessible dog populations across large states in a short period of time^20,29,30^. An economic study of ORV in dogs found that communities similar to Goa could likely implement this approach at a similar cost per dog vaccinated^31^.

According to the WHO criteria of cost-effectiveness^32^, the intervention can be considered “very cost-effective”. The cost of each year of healthy life (i.e., DALY averted) of 526 USD was a quarter of the gross domestic product per capita for India (2100 USD)^33^, and 13 times lower than that of Goa state (7029 USD)^34^ in 2019. The estimated cost per DALY averted from the Goa program was lower than statistical estimations of rabies interventional cost-effectiveness in India and Sri Lanka at 1064 USD and 1401 USD per DALYs respectively^35,36^, but higher than a recent report in Rajasthan at 40 USD per DALY averted^37^. This variability is likely a reflection of the non-standardization of economic modeling methodologies. However, unlike many assessments based on estimated values, state-wide implementation of the current program through a single government collaborator enabled comprehensive inputs to be based on programmatic operational and surveillance data. In the current analysis, we assumed that rates of PEP administration would not change until policies were updated to reflect the reduced risk and limited need for PEP. Assuming these changes are made in the lifetime of this program, the interventions would be even more cost-effective. The collaborative structure of the Goa program drove rapid innovation and expansion of activities, leveraging external funding to support the refinement of methodology and development of tools to increase efficiency, whilst the local government retained control and leadership of the intervention. Similar collaborative approaches were central to the global effort to eradicate polio^38^ and have been beneficial in rabies control initiatives elsewhere^39,40^. Proposed innovative funding structures, such as development impact bonds, may offer novel investment sources to stimulate the expansion of rabies control activities^41^.

The use of smartphone technology revolutionized the systematic, spatial management of the vaccination workforce in Goa^15^. A study in Haiti found that the same smartphone application used to spatially direct vaccination teams at the sub-village level, significantly increased vaccination coverage compared with non-technology-aided methods^14^. Such technology-aided vaccination strategies have the potential to minimize unvaccinated pockets in the population and therefore hasten viral elimination^42^. Similar strategies to spatially dissect national public health programs for delivery at the community level, described as “microplans”, have been reported as significant to the successful delivery of polio and other disease control interventions^43–45^. The availability of GPS location and dog-specific data for every animal vaccinated provided unprecedented transparency in reporting to government stakeholders in addition to a wellspring of insights into dog population ecology from which to optimize vaccination strategy.

The state-wide education initiative in schools directed rabies awareness towards children, the demographic at disproportionate risk of death from rabies^46^. Similar class structures, combining presentation, theater, and question-answer methods have been demonstrated to be effective in other settings^47^. Rabies educational content was integrated into the Government of Goa school science curriculum for children aged 11–12 years in 2020, helping to ensure sustained awareness of the disease whilst regional control efforts grow. The increase in dog-bite presentations at bite clinics during the project period may reflect the widespread community awareness of rabies brought about by the community education program and counters the traditional view that bite cases fall following rabies control^48^. Concurrent development of integrated bite case management (IBCM) systems to reduce unnecessary PEP in people bitten by healthy dogs would maintain cost-effective use of PEP in the face of this increase^49,50^.

Enhancing state capacity for the detection and diagnosis of rabies in dogs was critical to gaining insight into the scale of the problem; monitoring the impact of dog vaccination activities; and providing incentives for continued support from all stakeholders. In addition to these benefits, the removal of rabid dogs may also have hastened elimination by preventing ongoing rabies virus transmission^51,52^. Canine rabies surveillance focused on three main areas of enhancement: reporting, detection, and diagnosis. The Rabies Hotline proved to be highly effective in soliciting reports of suspect rabies cases from the public, police, animal welfare, human health, and veterinary sectors. Full-time Rabies Surveillance Officers capable of deploying to anywhere in the state at short notice ensured the timely veterinary investigation and testing of suspect rabid animals.

Prior to the local availability of OIE-approved rabies diagnostic tests, the use of rapid diagnostic tests motivated veterinarians to retrieve samples from suspect rabid animals under difficult field conditions^16^ and demonstrated the urgent need for in-state laboratory rabies diagnostic capabilities. Reports of poor quality control and low sensitivity of these tests, meant that negative results could not be considered valid and guidelines for their use should be a point of consideration in the development of national programs^53^. The establishment of DFA testing capacity in the government veterinary laboratory in 2016 was essential for robust state-level rabies surveillance and to ultimately demonstrate canine rabies freedom as recognized by OIE.

The use of portable MinION technology in Goa state revealed the potential for sequencing at regional veterinary laboratories to enhance state-level control strategies through a greater understanding of rabies virus transmission dynamics^17,54^. Time-scaled phylogenetic analysis of Goa sequences revealed a recent common ancestor around 2003–2005, with subsequent diversification into the three lineages, identified in this study as Goa1, Goa2, and Goa3. Further diversification of the Goa3 samples from the northern Goa border region around 2014, coincided with an increase in reported rabies cases in the area between 2014 and 2018. This finding supported the conclusion of the spatio-temporal cluster analysis indicating a potential site of the continued reintroduction of canine rabies. Expansion of dog vaccination beyond Goa’s borders is underway to reduce the risk of direct rabies virus reintroduction through dog movement at the border. The identification of a rabid dog importation into the greater region from northern India highlighted the risk of inter-state spread of rabies virus via human-mediated transport of dogs. National and regional rabies control will invariably require widespread coordination of vaccination efforts as was found to be critical to the success of programs in Europe and Latin America^55,56^. The continued monitoring of rabies virus sequences in Goa will provide a detailed picture of rabies virus transmission to further optimize control strategies on a larger scale.

The predominance of canine rabies in regions of high human and dog population density in Goa addressed the question of where efforts should be prioritized during the early phases of mass dog vaccination scale-up, when resources may be insufficient to vaccinate an entire state or district. The findings of the current study support an approach to accelerate the development of dog vaccination campaigns in Indian metropolis settings and the subsequent propagation of efforts into peri-urban and rural settings. This strategy was effective in regional rabies elimination efforts in Latin America, where nascent dog vaccination programs focused on large urban centers. These activities enabled the development of expertize, capacity, and political momentum to progress towards widespread initiatives ultimately resulting in the inter-state success of a magnitude needing to be replicated in India^20,56^. However, it is important to note that whilst urban centers may present the largest canine rabies burden, dog populations in peri-urban and rural regions can sustain rabies virus transmission^57^ and the limited access to PEP in rural communities often results in a disproportionate human rabies burden in this areas^3^. Further research is required to explore opportunities to spatially prioritize dog vaccination for maximum cost-efficacy of elimination programs.

National implementation of effective canine rabies control in India would represent the greatest achievement by a single country in the endeavor to eliminate dog-mediated human rabies by 2030. Clearly, this would require enormous mobilization of resources and sustained political commitment. Although the outputs of the current study would need to be amplified several hundred times over to be replicated at the national scale, it showcases the feasibility, cost-effectiveness, and considerable public health impacts of One Health interventions for rabies control at the state level in India. Enduring political support, modern technologies in program management, and intersectoral, transdisciplinary collaboration was pivotal to the success of this multi-year effort and provide a clear rationale for other states to follow. The growth trajectory of the Goa rabies control program aligned with that outlined previously by Wallace et al.^58^, with phases of preparation, scale-up, and sustained vaccination, resulting in rapid impacts on human and canine rabies incidence within three years of scale-up. Whilst dog accessibility presents a major challenge to achieving herd immunity in a predominantly unowned dog population, this study demonstrates that effective mass dog vaccination campaigns are feasible in India and can achieve canine rabies virus elimination across large geographic areas. In 2021, Goa became the first Indian state to be declared a Rabies Controlled Area^59^ under the Prevention and Control of Infectious and Contagious Diseases in Animals Act, 2009, ensuring legislation to maintain rabies control activities and setting a precedent for other states. The methods and technology developed through the current study can be leveraged to support the planning of national rabies control efforts in South Asia and accelerate similar examples of success in driving towards the 2030 goal of global dog-mediated human rabies elimination.

## Methods

The period of study was from 10/09/2013 to 31/12/2019, which coincides with the launch of the first pilot dog vaccination and education initiative and the end of the fifth year of large-scale vaccination activities. The Government of Goa Department for Animal Husbandry oversaw the project protocols and methods for mass dog vaccination and animal rabies surveillance, with input from the Goa Veterinary Association to adhere to all relevant ethical regulations. A veterinary ethical review was provided by the University of Edinburgh Veterinary Ethical Review Committee.

### Study site

Goa covers an area of 3700 square kilometers and has an estimated human population of 1.5 million, with 62% of people residing in areas defined as “urban”^60^. The state is bordered to the west by the Arabian Sea and to the east by the Western Ghats mountain range (Fig. 1). Tourism is a major industry. Goa is one of India’s more developed states, as measured by the United Nation’s human development index (HDI), with an HDI of 0.76 in 2017 as compared to India's national HDI of 0.64^61,62^. The state is divided administratively into two Districts, North Goa and South Goa, which are further divided into a total of 12 talukas. These talukas are made up of local administrative units of village panchayats (villages) and municipalities (towns and cities), of which there are a total of 420 in Goa.

### Dog vaccination

The non-governmental organization Mission Rabies (www.missionrabies.com) began investigation of mass dog vaccination methods in September 2013 with a two-week pilot vaccination campaign, followed by a dog vaccination and sterilization campaign in densely populated regions from March to September 2014. In September 2015, the Government of Goa established a formal collaboration through a Memorandum of Understanding with Mission Rabies to support the implementation of a state-wide dog vaccination initiative, rabies surveillance, and education program. The aim was to systematically vaccinate at least 70% of the dog population on an annual basis using a combination of DD and CVR methods (Supplementary Fig. 1)^2,9^. Vaccinations were provided free of charge and each dog was administered with a 1 ml dose of rabies vaccine (Nobivac® Rabies–MSD Animal Health) either subcutaneously or intramuscularly, depending on animal position and restraint method. Each dog was marked with non-toxic paint on the top of the head, lasting for several days to enable identification of vaccination status on post-vaccination surveys. Consent was obtained from an owner prior to vaccination of dogs that were identifiably owned.

Vaccination team direction and program monitoring were performed using the WVS App, a purpose-built mHealth technology described previously^15,26^. The information recorded offline for each dog at the time of vaccination included: vaccination team ID; time; date; GPS; sex; age; ownership; neuter status; confinement; and health status. A web-based interface enabled project managers to review daily the geographic extent of vaccination work and to spatially direct vaccination and survey teams to sub-village ‘Working Zones’ according to vaccination output^26^.

Between 2013 and 2017 dog vaccination was conducted entirely by CVR teams typically consisting of seven or more people traveling by truck: one vaccinator, one assistant, one driver, and four dog catchers using nets. Dogs that could be held by hand, either by an owner or the team, were manually restrained for vaccination. Dogs that could not be manually restrained were caught using nets (Supplementary Fig. 1). Two-person DD vaccination teams, consisting of a vaccinator and assistant traveling by scooter were introduced in 2018 to improve operational efficiency. DD teams vaccinated in Working Zones first, targeting dogs that could be restrained by hand and therefore did not require a large team of net-catchers. CVR teams subsequently followed in these regions to catch and vaccinate difficult to handle dogs that had not been vaccinated by DD teams, as indicated by the absence of a vaccination paint mark.

Post-vaccination dog-sight survey methods have been described previously and enabled immediate re-deployment of vaccination teams to Working Zones with low vaccination coverage^26^. In 2013 and 2014 only free-roaming dogs sighted were recorded, however, from 2015, dogs confined to private property at the time of sighting were also recorded. These surveys were performed following the completion of dog vaccination in each Working Zone to evaluate vaccination methodologies until 2017. In 2018 and 2019, post-vaccination surveys were used to spot-check coverage, and whilst vaccination teams were re-deployed to boost areas of low coverage, repeat surveys were not conducted as had been done in previous years. Therefore, surveys from 2018 and 2019 were not included in the analysis of vaccination coverage assessment.

### Rabies education

Alongside the state-wide systematic mass dog vaccination program, Mission Rabies implemented a concurrent education initiative focused on the delivery of structured lessons to children in schools and educational sessions to community groups. Typically, the education program was implemented through three rabies education officers who moved systematically across the state ahead of the vaccination schedule, delivering rabies lessons in schools and sessions to the community (Supplementary Materials)^15^. Rabies lessons were 15–30 minutes in duration, were adjusted to the age group, and fell under the following headings: Rabies is serious; Stopping dog bites; Rabies first aid; and Rabies is preventable^47^. In the later stages of the project, events to train schoolteachers in rabies lesson delivery were also conducted.

### Rabies surveillance

A central public rabies reporting and response service was established in March 2014, prior to which there was no structured process for the reporting of suspect rabid animals. A Rabies Hotline phone number was widely publicized to the public, government, and private sectors for the reporting of suspect rabid animals 24 hours a day, 7 days a week (Supplementary Methods, Supplementary Figure 1). Notification of a suspect rabies case to the Rabies Hotline triggered an immediate field investigation to examine the animal, and if necessary, submit samples for testing at the Goa Disease Investigation Unit (Supplementary Methods). The Rabies Hotline and rabies response activities were not active from October 2014 to September 2015. Canine rabies surveillance intensity increased in April 2018 through the incorporation of IBCM methods described elsewhere^50^. Suspected human rabies cases were managed and diagnosed at the Goa Medical College, with numerous events and initiatives taking place during the study period to raise awareness of the importance of human rabies surveillance in the medical profession (Supplementary Methods). PEP was available free of charge to those presenting for treatment of dog bites at government medical facilities throughout the state.

### Data analysis

Data for dog vaccinations, educational events, post-vaccination dog surveys, notifications of suspect animal rabies cases and suspect animal rabies case investigations were exported from the WVS App database in CSV format. Analysis was performed using R version 3.6.2. Manual reports and field records were used to verify App data. Post-vaccination surveys and dog vaccination data were used to calculate mean dog vaccination coverage and month-wise vaccination coverage by taluka (Supplementary Methods).

### Logistic regression model

A mixed-effects multivariable logistic regression model was used to identify factors associated with a taluka having at least one confirmed dog rabies case each month (Supplementary Software 1). The number of confirmed dog rabies cases for each taluka each month was used to create the binary outcome variable in the model. If a taluka had at least one case in a given month it was considered positive, and if no cases were recorded that month the taluka was considered negative. Explanatory variables investigated included free-roaming dog population, free-roaming dog population density, estimated monthly vaccination coverage, estimated 12-month rolling mean coverage, season, and whether the taluka borders unvaccinated dog regions (Supplementary Data 1). The seasonal timeframes used have been described previously^63^. The data were randomly split into a training and testing dataset using a 70:30 ratio using R package caret^64^. Univariable analysis was used and any variable with a *p* value of <0.15 was considered for the final model. To investigate whether numerical variables had a linear relationship with the log-odds of the outcome, these were split into quartiles and univariable models were visualized to assess the relationship. Manual forward variable selection was conducted and the final model was chosen based on the lowest Akaike information criterion (AIC). The final model was validated, testing its ability to predict the outcome in the test dataset by estimating the area under the curve using R package ROCR^65^.

### Phylogenetic analysis

Rabies viral sequencing of glycoprotein and nucleoprotein genes was performed on an archive of positive canine rabies brain samples spanning 2016 to 2018 using a MinION sequencer (Oxford Nanopore Technologies) at the Goa Disease Investigation Unit (Supplementary Methods). Phylogenetic analysis was performed to evaluate the similarity between samples and to compare them with historic references^17^ (Supplementary Methods).

Time-scaled phylogenies were generated from complete nucleoprotein and partial glycoprotein gene (1317 nt) data using BEAST v1.10.4 and GTR + G + I substitution model with two partitions (1 + 2 and 3) and an uncorrelated relaxed lognormal molecular clock. Years of sample collection were used as tip dates, with an uncertainty of 1. The mean of the clock rate prior was set to 10^-4^ (normal distribution, SD = 1). Both analyses lacked calibration data points and estimated most recent common ancestor ages should be considered relative and are highly influenced by the samples included, which was biased toward recent samples due to improved access to sequencing. Maximum clade credibility trees were generated using TreeAnnotator and visualized in FigTree v1.4.4. Map of India was generated from shapefile data at GADM.org using raster, ggspatial, and ggplot2 packages in RStudio (R v4.0.2). Figures were finished in InkScape. Reference sequences included in the time-scaled phylogenetic analyses were chosen based on sequence length (full-length nucleoprotein gene and at least 1317 nt of glycoprotein gene), isolation in India or neighboring countries, inclusion in Arctic-like 1a lineage, year of isolation available, and sequence quality. Sequences published by Deventhiran et al. (2018)^66^ were not included in these analyses as several sequencing errors were observed and phylogenetic clustering of nucleoprotein and glycoprotein gene sequences from that study was inconsistent. Arctic rabies virus variant sequences KX148105 and JQ148105, Arctic-like 3 sequence KX148228, and Arctic-like 1b sequences KX148225, KX148226, HE802676, KX148227, KF150745, KF150744, and MK760761 were used as outgroups^67^.

### Spatio-temporal cluster analysis

A space-time scan statistic was used to detect statistically significant spatio-temporal clusters of canine rabies using a discrete Poisson retrospective space-time probability model^68^ as used in a number of other studies of rabies distribution^69,70^ (Supplementary Software 2). For the purposes of this analysis, the village was considered the study unit, and canine rabies cases recorded each month were aggregated at the centroid of each village for use in the model. The human population of each village was used as a proxy for the population at risk. Cluster analyses were performed using SaTScan™ v9.6 software^71^ through R package rsatscan^72^. Satscan uses Monte Carlo hypothesis testing to obtain the *p* values. For this analysis, we used 9999 Monte Carlo replications, and a cluster was considered statistically significant if the *p* value was <0.05. The maximum size for the mobile window of the scan was set as 20% of the population at risk with a circular shape.

### Cost per dog vaccinated

In-country annual program expenditures were used alongside annual dog vaccination output to estimate the cost per dog vaccinated. This expenditure was determined from account records reporting on Goa expenditure on all sources of income, including government and charitable grants, and the estimated value of donated vaccine (Supplementary Tables 3–5). A breakdown of dog vaccination program expenditures, within total program expenditures, was only available for 2018 and 2019 (Supplementary Table 5).

### Cost-effectiveness model

The RabiesEcon model, developed by the US Centers for Disease Control and Prevention, was used to estimate the impact and cost-effectiveness of the intervention (Supplementary Data 3)^18,19,73^. Goa values were inputted into the RabiesEcon version used by Kunkel et al.^18^ to assess the additional costs and benefits of the Goa rabies control program as compared to no intervention. Input values from Goa, including human and dog population data, cost of dog vaccination and PEP, annual dog vaccination output, and estimated rates of access to PEP during and after the program (Supplementary Data 3). Predicted outcomes such as estimated human exposures and human rabies deaths were cross-checked with real-world data. The RabiesEcon model was published by Kunkel et al. (2021) was modified to account for costs associated with PEP administration resulting from exposures to animals that were unavailable for diagnostic testing. This adjustment was made by including a feature to allow the user to estimate the reduction in PEP after the implementation of the rabies control program. The equation in the tab “Data”, row 27 was modified to reflect the larger of either the pre-program costs multiplied by the user-defined PEP reduction rate or the number of true rabies virus exposures estimated by the model. As part of the data inputs to the model, it was assumed that there was no reduction in PEP administration during or after the intervention. All annual costs were discounted at 3%. The costs associated with surgical sterilization of dogs by non-governmental organizations were not considered in the model.

### Reporting summary

Further information on research design is available in the Nature Research Reporting Summary linked to this article.

## Supplementary information

Supplementary Information

Description of Additional Supplementary Files

Supplementary Data 1

Supplementary Data 2

Supplementary Data 3

Supplementary Software 1

Supplementary Software 2

Reporting Summary

## Data Availability

Data supporting the findings of this work are available within the paper and its Supplementary Information files. All sequences generated as part of this study are deposited in GenBank (accession codes: MW054945–MW055041). Source data are provided in this paper.

## Source data

Source Data

## Code availability

The R code used in the logistical regression and spatio-temporal analysis is included in the Supplementary Files.
