# Supplementary material for: Elimination of human rabies in Goa, India through an integrated One Health approach
Source: Nat Commun. 2022 May 19;13:2788. doi: 10.1038/s41467-022-30371-y (PMC9120018; doi:10.1038/s41467-022-30371-y)
Supplement: Supplementary file 1 — Supplementary Information [file 41467_2022_30371_MOESM1_ESM.pdf]

Supplementary Information

Elimination of human rabies in Goa, India through an integrated One Health approach

Contents

Supplementary methods 2

Education campaign 2

Canine rabies surveillance 2

Human rabies surveillance 3

Dog vaccination 3

Vaccination coverage estimates 3

Phylogenetic analysis 4

Supplementary Figures 6

Supplementary Tables 21

Additional supplementary files 27

Supplementary References 28

Supplementary methods

Education campaign

School rabies lessons comprised a presentation in the local language, incorporating visual, auditory, and kinesthetic teaching styles through theatre, demonstration and question-answer activities. Lessons were delivered to groups, from the class level to the entire school, dependent on the school's preference, schedule, and facilities. Data about schools attended, teacher-training sessions and community events were recorded in the WVS App following delivery, including time, date and GPS location of the entry, the number of children, adults or teachers educated and the type of lesson.

Canine rabies surveillance

The Rabies Hotline was publicised to the public, police, veterinary services, health centers, local administrative authorities and NGOs through education activities, newspaper articles, online media and door-to-door vaccination teams. Rabies Hotline cards were distributed by vaccination teams and at education events (Supplementary Fig. 1). Calls were screened to evaluate the history and presenting signs for risk of rabies in the dog, including aggression, hypersalivation, ataxia, neurological signs, collapse, and sudden death. The digital recording of call records began in October 2017 through customized forms in the WVS App.

If rabies was suspected, a response team was deployed to investigate the case. Initially the response teams consisted of a veterinarian and a dog catching team, however the protocol was adjusted in 2018 with the introduction of integrated bite case management (IBCM) methods[1]. The investigation was conducted by a trained, full-time Rabies Surveillance Officer who called for additional support if a net-catching team was required to restrain a rabid animal. Each investigation included assessment of the animal and interviews with community members to identify exposed people and animals. If an animal showed signs of rabies, it was removed and transported to a secure location for further monitoring. When a veterinarian evaluated the animal as showing signs of rabies and suffering, it was humanely euthanized in accordance with the Animal Welfare Board of India advisory and the Prevention of Cruelty to Animals Act 1960[2].

Historically, rabies diagnosis was performed at the Goa Disease Investigation Unit by identification of Negri bodies in Sellers-stained brain tissue. From March 2014, Anigen lateral flow assay (LFA) tests (Anigen Rapid Rabies Ag Test Kit, Bionote, Hwaseong-si, Korea) were performed in the field at the time of post-mortem[3]. Frozen brain samples were batched for transportation to the National Institute for Mental Health and Neurosciences (NIMHANS) some 600km away, for diagnosis using the direct fluorescent antibody (DFA) test approximately once a fortnight. State diagnostic capacity for rabies case detection was established in December 2016 through the donation of a fluorescent microscope to the Disease Investigation Unit (DIU) in the Department of Animal Husbandry and Veterinary Services, enabling more timely rabies diagnosis. NIMHANS provided DFA proficiency testing on an annual basis from 2017 to 2019 on 10 randomly selected stored samples from throughout the year to evaluate concordance with DFA test results from the Goa DIU.

Human rabies surveillance

All suspect human rabies cases in Goa state were routinely transferred to Goa Medical College (GMC), a tertiary medical hospital. Human rabies incidence was monitored through GMC records. Data on human rabies deaths were provided by the Directorate of Health Services (DHS), as submitted to the India National Rabies Control Program. Diagnosis was based on the DFA test using brain samples at NIMHANS, Bangalore.

The launch of the National Rabies Control Program (NRCP) by the National Centre for Disease Control, in December 2016 brought renewed funding for training in human rabies diagnosis and PEP to medical practitioners throughout government hospitals in Goa. State-level rabies engagements with the medical profession included a sampling and diagnosis workshop conducted at GMC in December 2017 through a collaboration between the DHS, GMC, Mission Rabies and NIMHANS. The workshop was attended by residents and faculty of the Department of Medicine, Pathology, Pediatrics, Psychiatry, Forensics and Neurology. GMC also hosted the Association for the Prevention and Control of Rabies in India (APCRI) national rabies Conference in GMC in July 2017. In April 2018 the Department of Animal Husbandry and Veterinary Services hosted the state level Stepwise Approach to Rabies Elimination (SARE) Workshop in collaboration with the U.S. Centers for Disease Control and Prevention (CDC), bringing together human and animal health stakeholders and emphasising the importance of human rabies diagnosis.

Dog vaccination

Dog vaccination program outputs were calculated from individual dog vaccination records in the WVS App. The number of active vaccination teams per day was calculated using entries per unique user identity (i.e., vaccination team) per day to identify active vaccination teams. Team outputs of less than 10 vaccinations on a given day were not considered as these were likely to be entries on off-days or opportunistic vaccination, instead of true working vaccination days.

Vaccination coverage estimates

Estimated coverage was calculated from post-vaccination dog sight surveys conducted after vaccination in each Working Zone. Where multiple surveys were performed of the same Working Zone during the same vaccination cycle, the last survey performed was used for estimation of final vaccination coverage. Estimated vaccination coverage was calculated using the formula:

$$Vaccination\ coverage = \frac{Dogs\ with\ mark\ sighted}{Total\ dogs\ sighted}$$

The timing and extent of post-vaccination surveys was altered in 2018 and 2019 compared to previous years, in that not all areas vaccinated were surveyed and, in some cases, additional dogs were vaccinated in Working Zones after the completion of the final post-vaccination survey (Supplementary Fig. 5, Supplementary Fig. 6). As a result, the estimated vaccination coverage would not reflect the final dog vaccination coverage.

The dog population for each taluka was estimated by campaign period due to the geographic cycle of vaccination and survey work crossing the January-December calendar year in some talukas (Supplementary Fig. 2). Population estimates were only performed for campaign periods in which post-vaccination surveys were routinely conducted (Supplementary Figs. 6). The taluka total dog population

was estimated for a particular campaign using the mean vaccination coverage from post-vaccination surveys and total dog vaccinations applied to Lincoln—Petersen's formula[4] (Supplementary Fig. 7). The mean campaign dog population estimate was used as an approximation of the taluka dog population throughout the study period.

The taluka month-wise point vaccination coverage was approximated as the estimated immune dogs in the population that month divided by the total dog population estimate. The month-wise number of immune dogs in the population was estimated as either the number of immune dogs from the previous month reduced by a factor of population turnover, or in months in which a vaccination campaign took place, the total number of dogs vaccinated during the campaign. Because each campaign vaccinated the population without consideration of each dog's previous vaccination status, the estimated total number of immune dogs was reset where the campaign total vaccinations exceeded the current estimated number of immune dogs, as described in the following equations:

$$R_m = 0.94(I_{m-1}) \tag{1}$$

$$I_m = \begin{cases} C_m, & if\ C_m \geq R_m \\ R_m, & if\ C_m < R_m \end{cases} \tag{2}$$

$$Prop_m = \frac{I_m}{D} \tag{3}$$

The I_m was the estimated number of immune dogs in the population for a particular month (m). R_m was the estimated remaining number of immune dogs in the population calculated from the immune dogs the previous month after application of a decay factor to account for estimated monthly population turnover (0.94). C_m was the number of new dogs vaccinated in a campaign centred on the campaign month of maximum vaccinations. $Prop_m$ was the estimated vaccination coverage in the population for a particular month. D was the estimated dog population for each taluka. As outlined in equation 2, where the number of campaign vaccinations did not exceed R_m, the campaign was not included in calculation, as these were erroneous vaccination entries not under a particular campaign (Supplementary Fig. 12).

Phylogenetic analysis

Viral sequencing was performed on a MinION sequencer (Oxford Nanopore Technologies), as described by Gigante et al.[5]. Coding regions were aligned using the FFT-NS-ix1000 algorithm in Mafft v.7.308[6] in Geneious 9.1.4 (Biomatters, Inc., Newark, NJ, USA). Phylogenetic analysis was performed based on maximum likelihood in Mega7[7] with 1,000 bootstrap replicates using all sites. GTR+G+I model was chosen based on modeltest in Mega 7. Trees were rooted using bat rabies virus lineage sequences JQ685901 and JQ685945. Reference sequences were used to show the phylogeny of the following rabies virus lineages: Arctic (JQ944707, KX148105), Cosmopolitan (JQ944708, JQ944706, JQ944705), Arctic-like 1b (KX148225, HE801587, JX987739, KF150744, MK760761, KX148226, HE802676), Arctic-like 2 (KC171645, KC171644, KC171643, KM272192, GU937029), and Arctic-like 3 (KX148228). The following criteria were used for preliminary assignment of samples to groups based on nucleotide identity of the

rabies virus glycoprotein coding region: a sequence must display >99% nucleotide identity with all other sequences within the group and <99% nucleotide identity with sequences in other groups; and a group was required to contain more than one sequence. Haplotype network analysis was performed in popart[8] using the Median Joining Network method[9] (epsilon = 0). The Goa map was made in RStudio (R version 3.6.1) using raster[10], ggplot2[11] and ggspatial[12] packages and shapefile data from the Database of Global Administrative Areas (GADM)[13].

Supplementary Figures

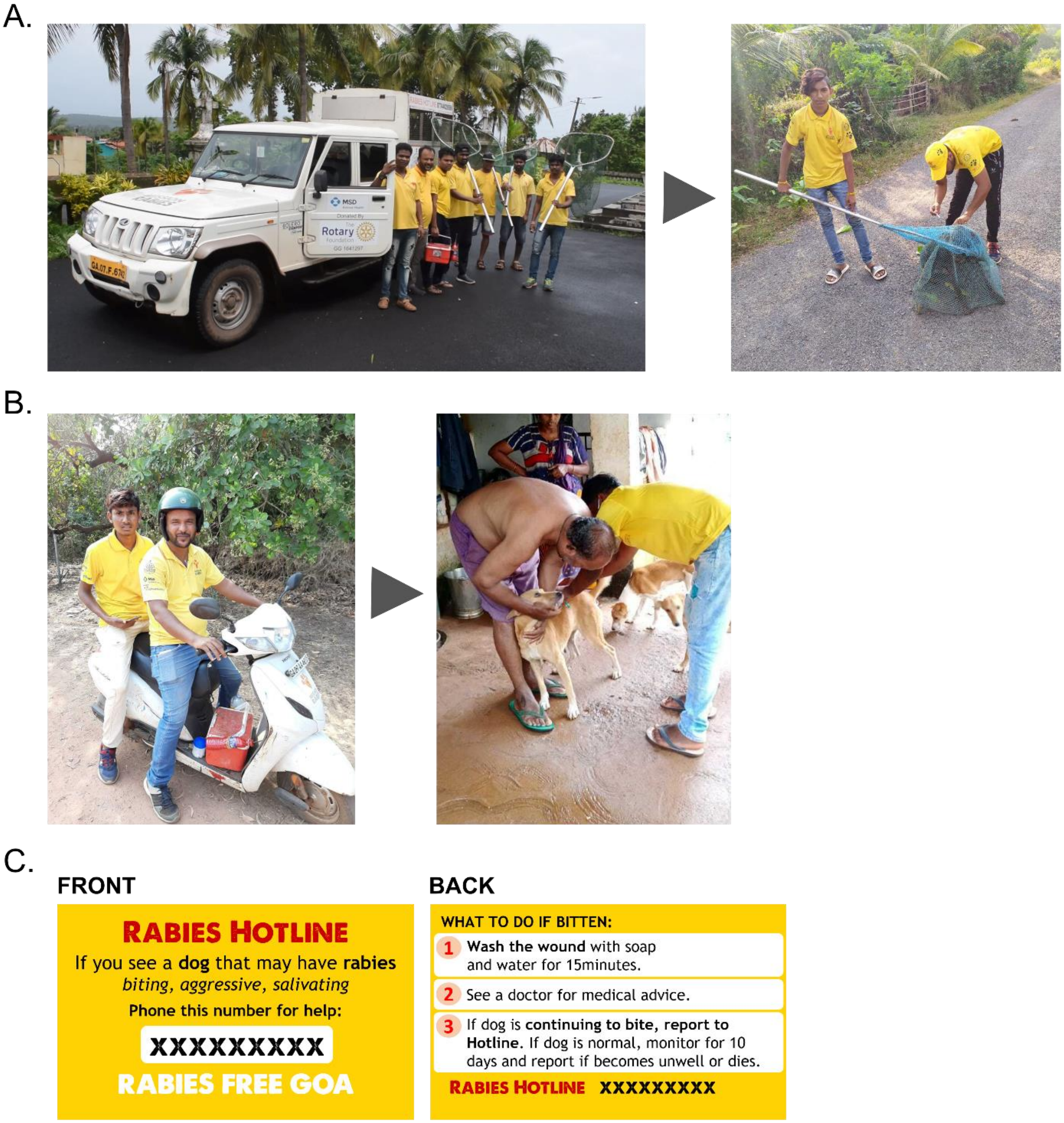

Supplementary Figure 1 – Dog vaccination methodology and Rabies Hotline card.
A *Capture-vaccinate-release (CVR) teams consisted of seven or more people travelling by truck through communities predominantly focusing on stray dogs. Dogs were caught for vaccination either by hand or using nets.* ***B*** *Door-to-door (DD) vaccination teams consisted of two people travelling through communities by 2-wheeler scooter, approaching dog owners house-by-house requesting them to present dogs for vaccination and restraining dogs manually for vaccination.* ***C*** *Information shown on Goa Rabies Hotline cards of a convenient 'business card' size for keeping in a purse or wallet (5.1cm x 8.9cm). These cards were widely distributed by vaccination teams and rabies educators during all activities to promote reporting of suspect rabies cases. Logos, graphics, and the specific phone number have been removed for reproducibility. All identifiable persons photographed gave permission for use of images.*

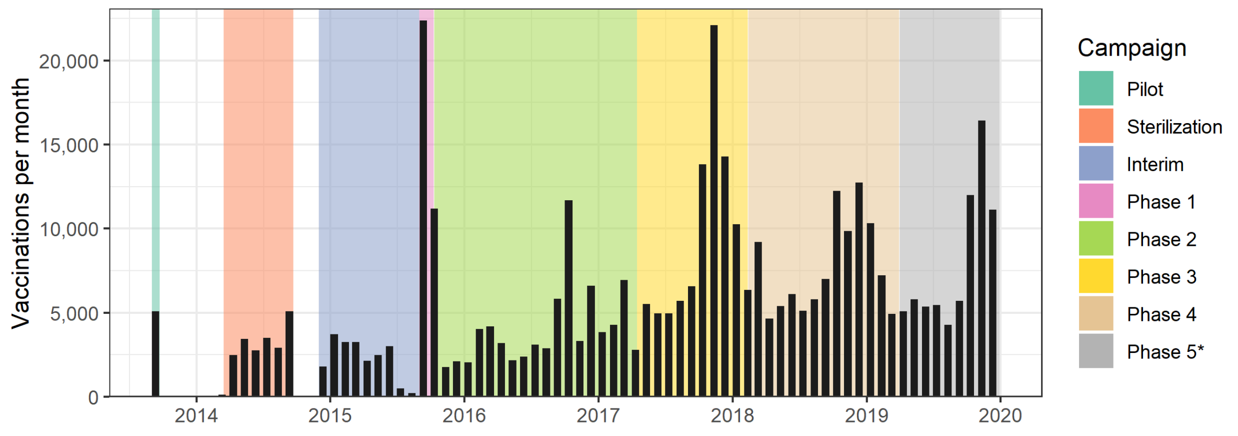

Supplementary Figure 2. Monthly dog vaccinations against rabies by campaign period.
*Total vaccinations per month during the study period. For the most part the geographic vaccination cycle repeated on a 12-monthly basis, moving from taluka-to-taluka in roughly the same order to achieve once yearly vaccination of each area. These cycles are denoted by background colors in the plot. Cycles prior to Phase 3 vaccinated the dog population to differing intensities and geographic extents as methods were refined. Phases 3,4 and 5 represent state-wide intensive dog vaccination (Fig. S3). *Phase 5 campaign was ongoing at the end of the study period.*

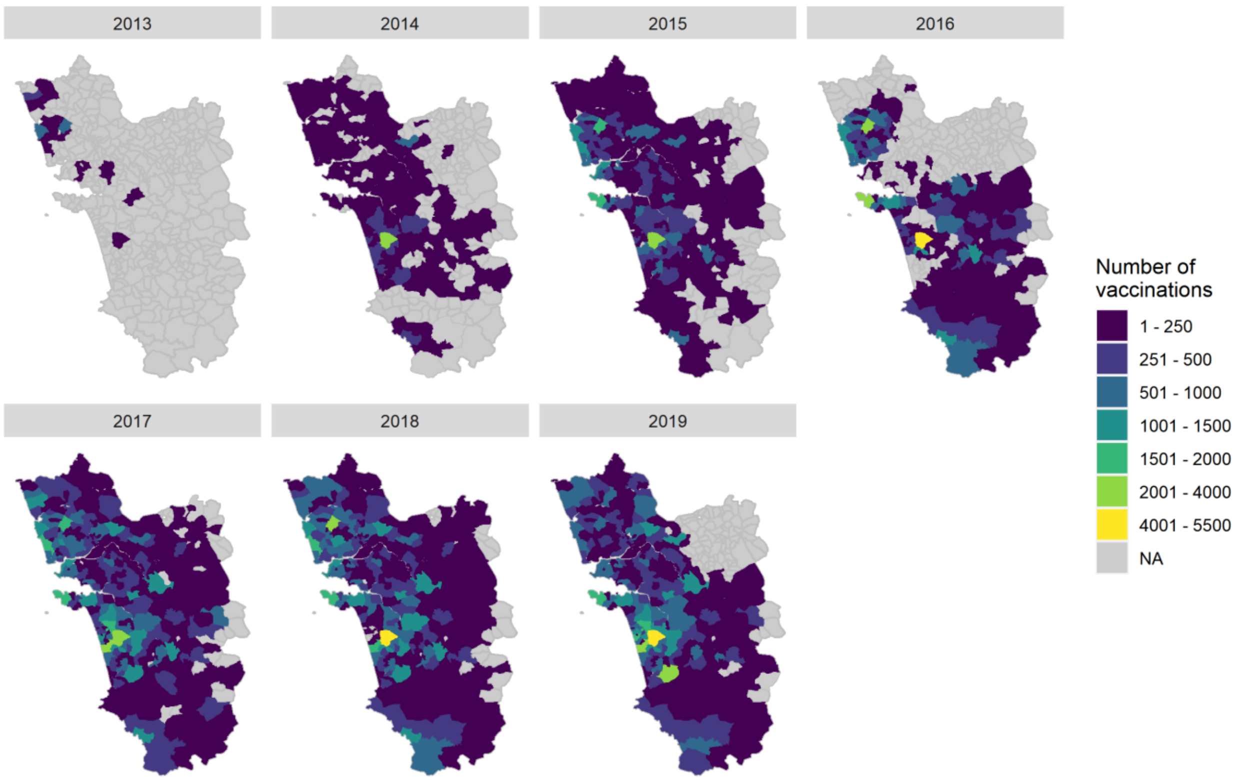

Supplementary Figure 3. Geographic extent of dog rabies vaccination by year.
Choropleth map of Goa showing total doses of vaccines delivered by village/municipality region by year from 2013 to 2019. Grey regions indicate no vaccinations.

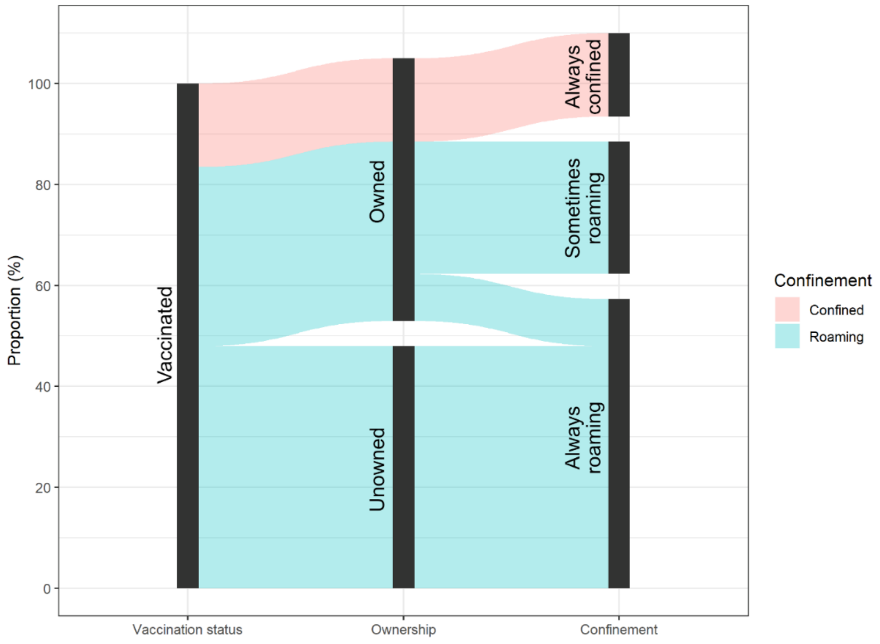

Supplementary Figure 4. Distribution of dog ownership and confinement within the vaccinated population.
Parallel set plot showing the distribution of ownership and confinement details for all dogs vaccinated during the study period for which confinement status was recorded (n = 384,238).

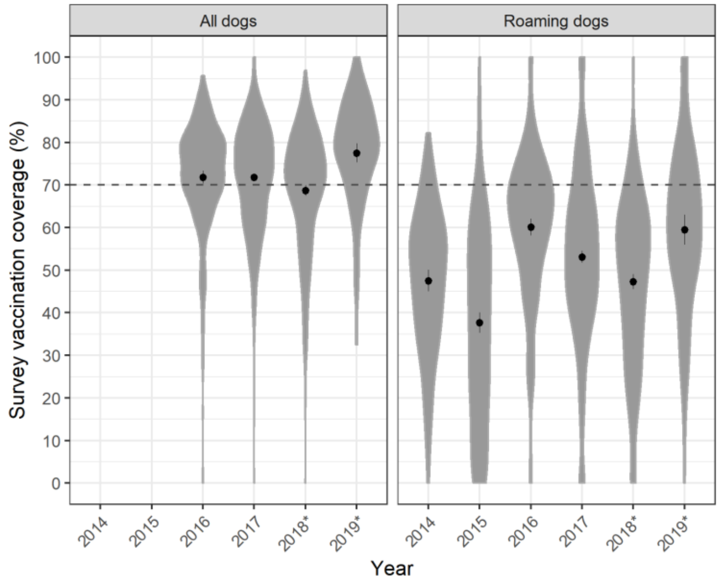

Supplementary Figure 5. Mean vaccination coverage by year.
Mean vaccination coverage from final zone-level post-vaccination surveys by year in total dog population and roaming dogs sighted. Data collected during 2014 and 2015 were only based on roaming dogs. Number of surveys by year: 2014 n = 168; 2015 n = 360, 2016 n = 323, 2017 n = 847, 2018 n = 512, 2019 n = 125. The mean vaccination coverage is indicated by black dots. Error bars indicate 95% confidence limits, calculated using a 2-sided t-test. Grey shaded area indicates the distribution of all post-vaccination survey vaccination coverages. Asterisk indicates that the post-vaccination survey extent and timing followed a different methodology in 2018 and 2019 as compared to previous years. In 2018 post-vaccination surveys were conducted and vaccination repeated where coverage was low, however a final survey was no longer routinely performed following repeat vaccination, which could result in an underestimate of the final vaccination coverage achieved. In 2019 the geographic extent of Working Zones surveyed was markedly reduced due to operational constraints (Fig. S6).

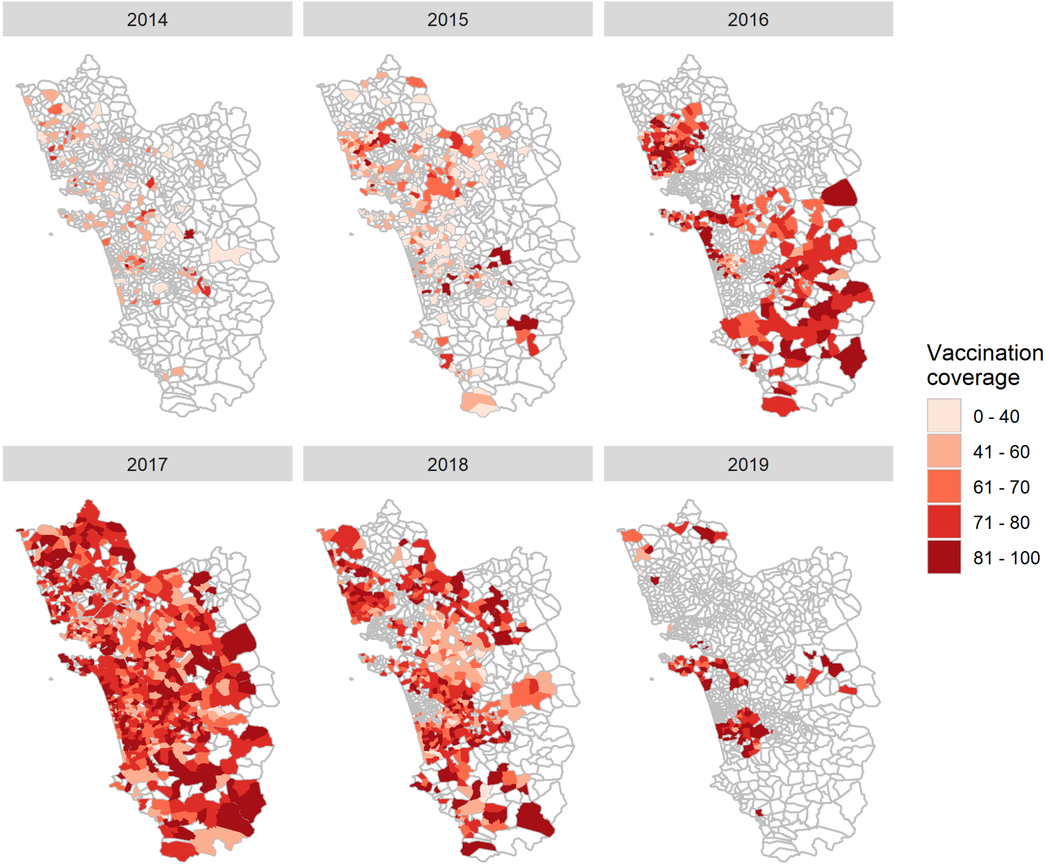

Supplementary Figure 6. Vaccination coverage from post-vaccination surveys by Working Zone from 2014 to 2019.

Choropleth map of Goa showing distribution of final post vaccination surveys by Working Zone regions and estimated vaccination coverage in all dogs sighted from 2014 to 2019. Only free-roaming dogs were recorded on surveys in 2014 and 2015, whilst maps for 2016 to 2019 show all dogs sighted, including those confined to private property at the time of sighting. The timing and extent of post-vaccination surveys was altered in 2018 and 2019 as compared to previous years meaning that not all areas vaccinated were surveyed and additional dogs may have been vaccinated in Working Zones after the completion of the final post-vaccination survey.

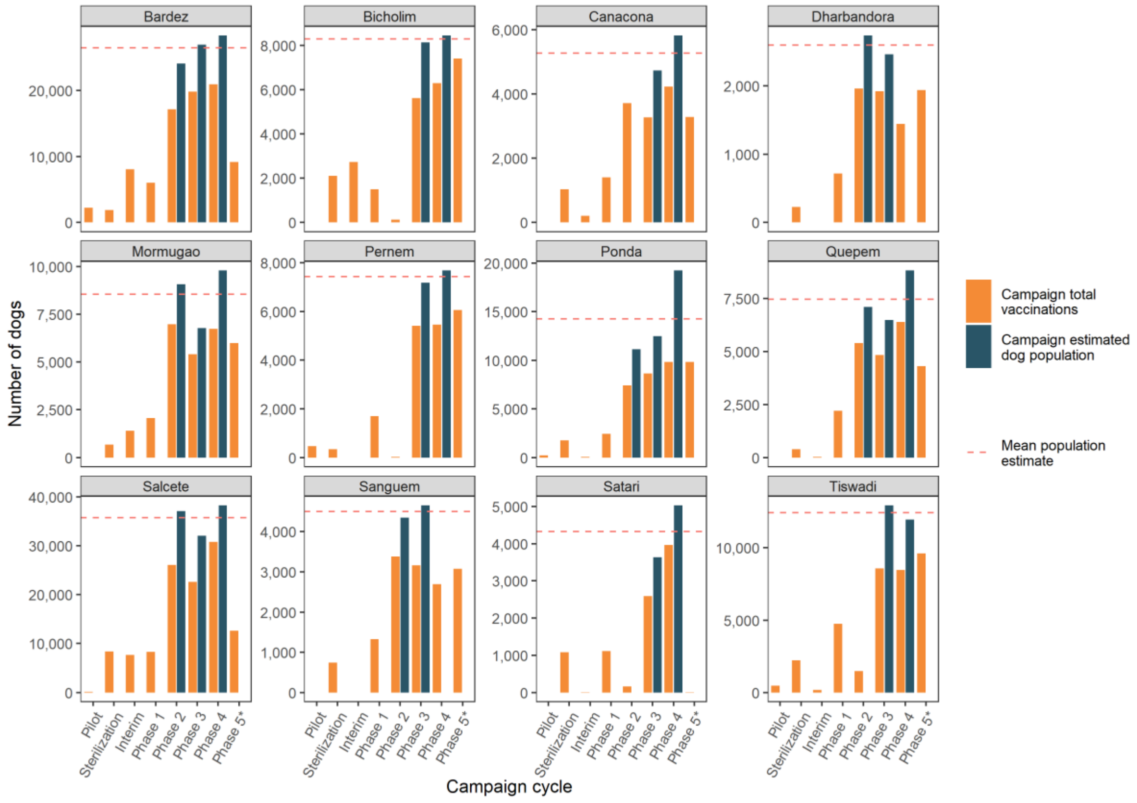

Supplementary Figure 7 – Dog population estimates by taluka.

*Graphs showing total vaccinations per taluka by campaign period (Supplementary Figure 2). Total dog populations were estimated for campaign periods where routine post-vaccination surveys were conducted. The total dog population was estimated using the taluka mean campaign vaccination coverage and total dogs vaccinated, applied to Lincoln—Petersen's formula. The horizontal red dotted line shows the taluka mean dog population estimate across campaigns. *Phase 5 vaccination was ongoing in Salcete and Satari at the end of the study period.*

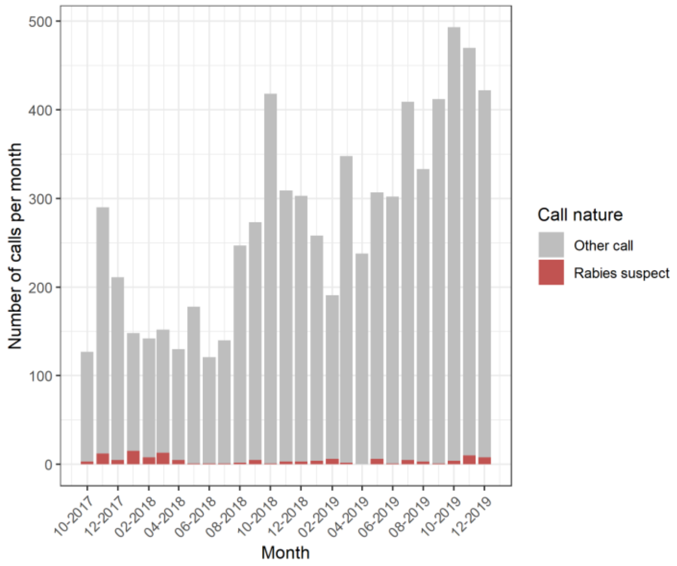

Supplementary Figure 8. Rabies Hotline calls by month and outcome.
Chart of total calls received to the Goa state rabies Hotline from October 2017 to December 2019. Red bars indicate the number of calls where the report was considered to be a potential sighting of a rabies case and warranted further investigation.

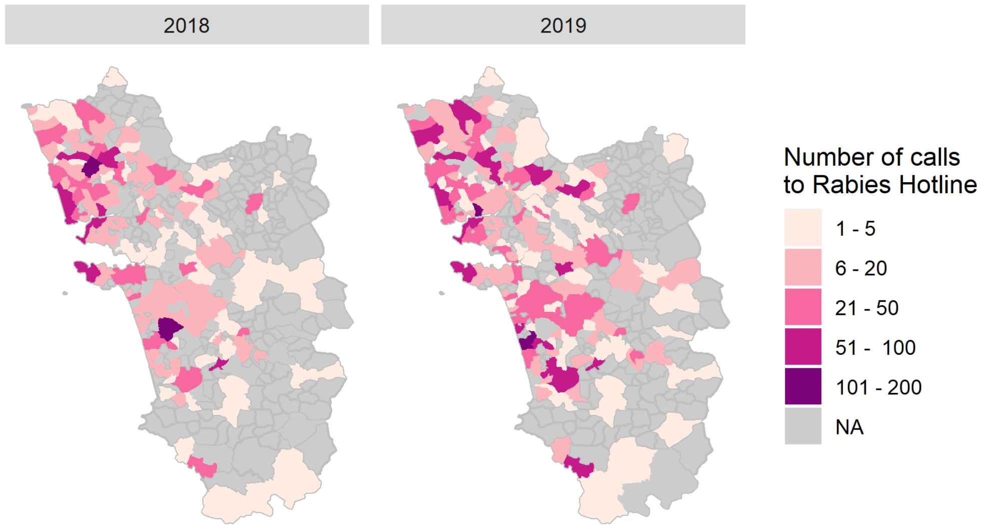

Supplementary Figure 9. Origin of calls to Rabies Hotline in 2018 and 2019
Choropleth map of Goa villages showing the origin of calls to the Rabies Hotline in 2018 and 2019.

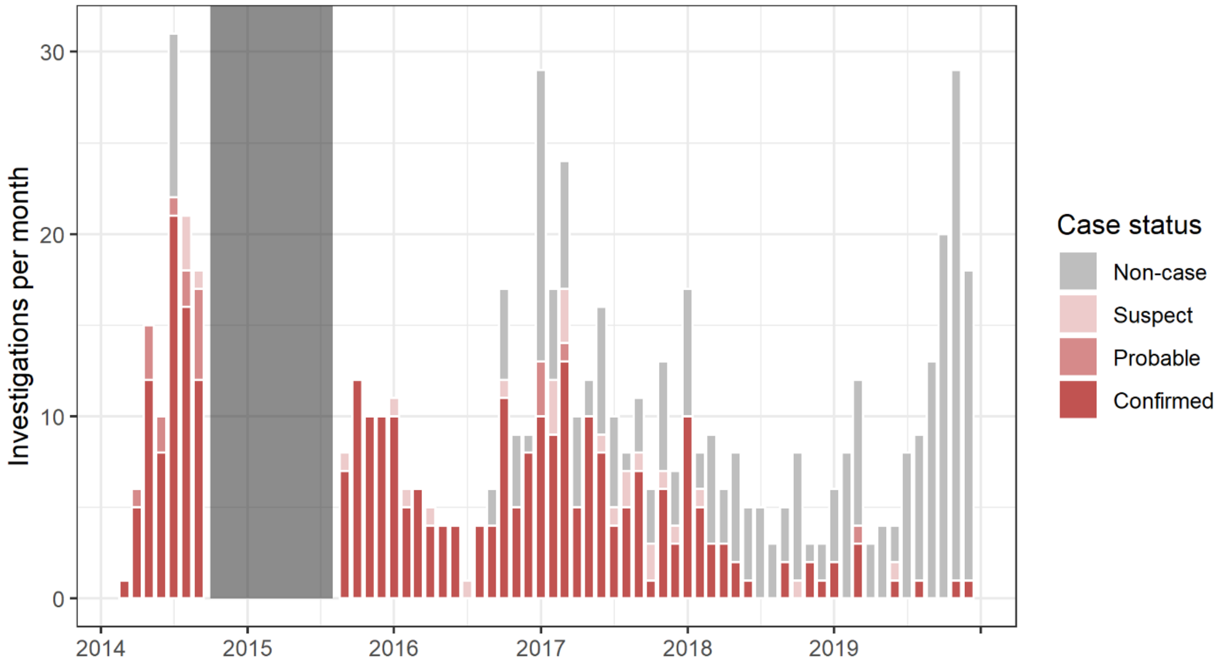

Supplementary Figure 10. Monthly canine rabies case incidence (2014 – 2019).
Chart showing the outcome of laboratory tests of suspect animal rabies cases by month, colored by outcome status. The dark grey box denotes a period where surveillance systems were not active.

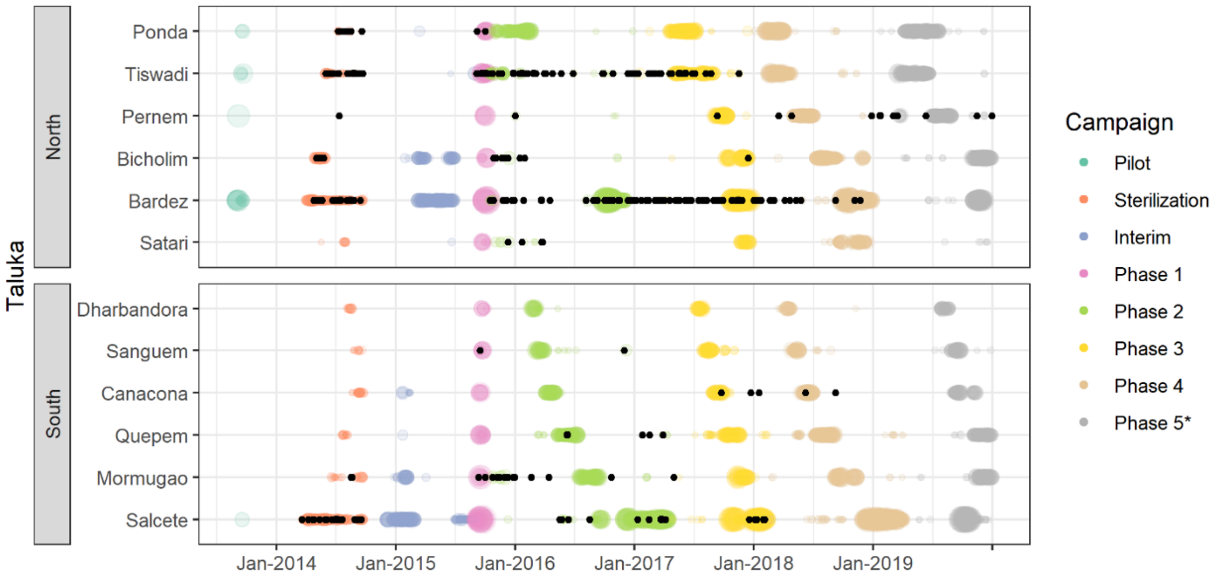

Supplementary Figure 11. Taluka-wise rabies case and dog vaccination occurrence.
*Timeline of dog vaccinations by campaign (colored circles) and confirmed canine rabies case detection (black dots) by Taluka from 2014 to 2019. *Phase 5 campaign was ongoing at the end of the study period.*

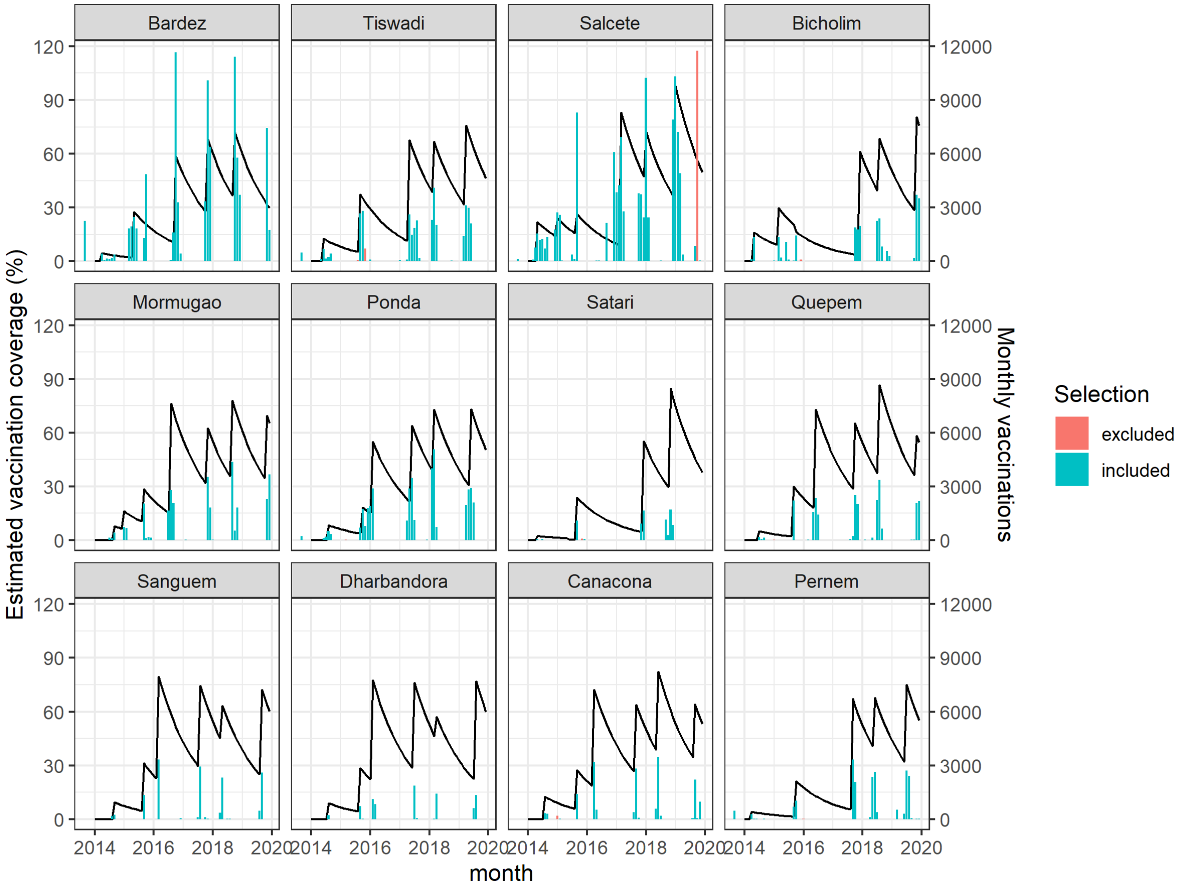

Supplementary Figure 12. Monthly dog vaccinations by taluka and point-vaccination coverage estimate.
Graph showing estimated vaccination coverage by region (black line, left y-axis) and total vaccinations (bars, right y-axis), colored by whether the vaccination figure was included in calculation of point-vaccination coverage. Due to each campaign vaccinating the population afresh, without consideration of individual animal's vaccination history, campaigns were excluded where the number of campaign vaccinations did not exceed the estimated number of dogs vaccinated in the population at that time point. A notable example is the 2019 Salcete campaign due to this campaign spanning the New Year and therefore not reaching peak vaccination coverage at the end of the study period.

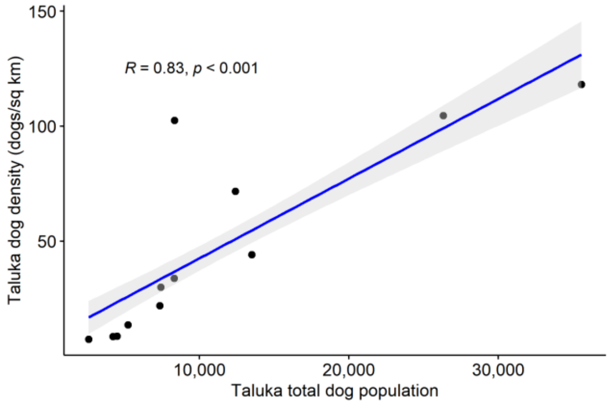

Supplementary Figure 13 – Relationship between estimated total dog population and dog density for the talukas of Goa.

Scatter graph showing the estimated total number of dogs and dog density per taluka. Created using R package, ggscatter[14]. The blue line shows the linear regression line, with 95% confidence interval (grey shading). R is the Pearson correlation coefficient with associated p-value (p = 2.15e-19), which indicates high positive correlation.

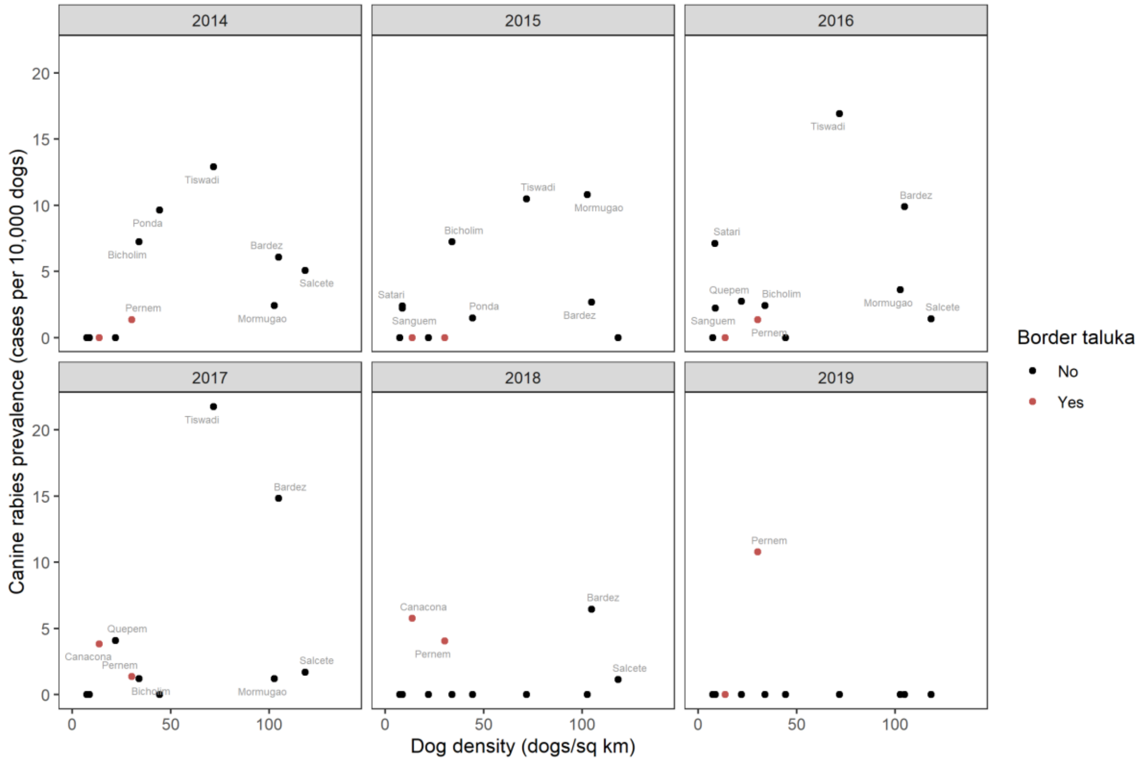

Supplementary Figure 14 – Annual canine rabies prevalence in relation to estimated dog density for the talukas of Goa.

Scatter plots of estimated taluka dog density and annual canine rabies prevalence per 10,000 dogs from 2014 to 2019. The progressive increase in dog vaccination effort by taluka over the course of the study period (2014 – 2019) is not indicated in this plot, however, can be seen in other figures. Canine rabies prevalence was calculated as the number of positive rabies cases per taluka per year, divided by the estimated taluka dog population and presented as cases per 10,000 dogs. The colour of the points denotes talukas at the north and south borders of Goa, near high density regions of neighbouring rabies endemic states. The name of talukas is labelled for points where the canine rabies prevalence is greater than zero.

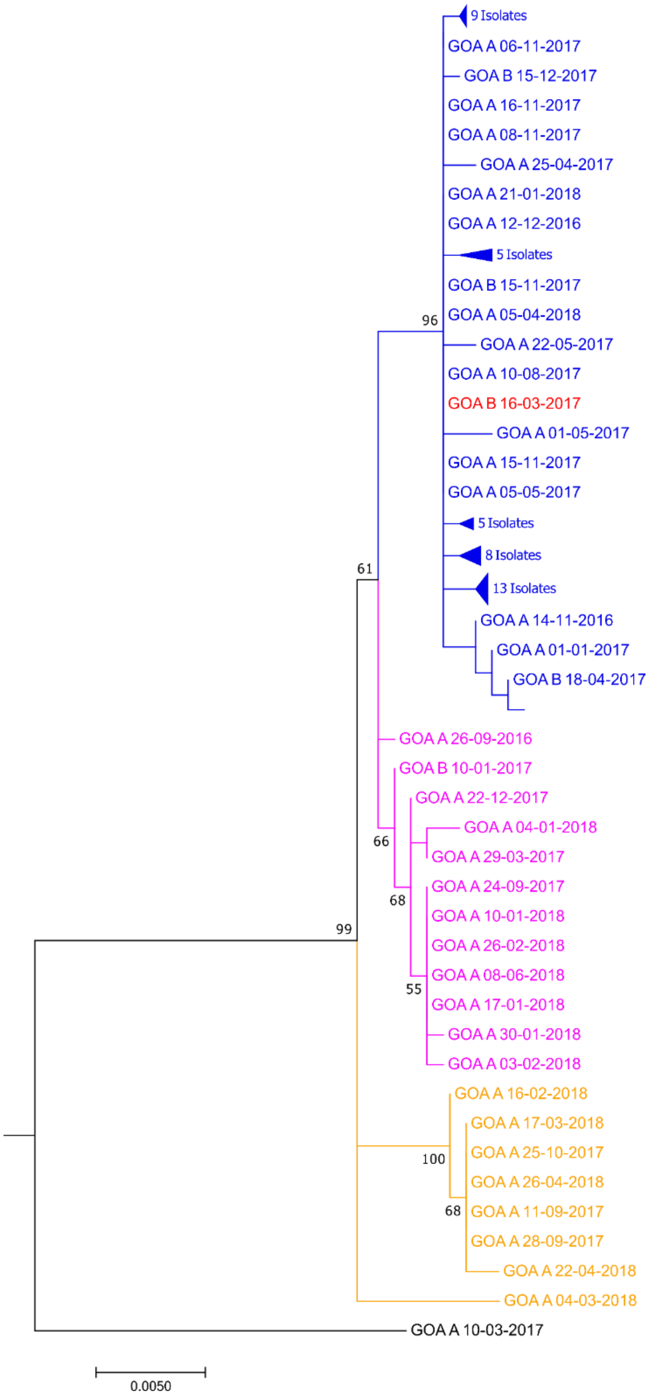

Supplementary Figure 15. Rabies virus nucleoprotein gene sequences.

Phylogenetic analysis of 80 rabies virus nucleoprotein gene sequences from Goa, India 2016-2018. Phylogenetic tree calculated by maximum likelihood method (GTR+G+I). Numbers on branch points represent bootstrap support based on 1000 replicates. Tree colors correspond to grouping (Blue Goa1, Magenta Goa2, Yellow Goa3). Some sequences in Goa1 are collapsed for viewing convenience; a list of samples in each group can be found in Supplemental Table. Discrepant sample GOA_B_16-03-2017 is shown in red.

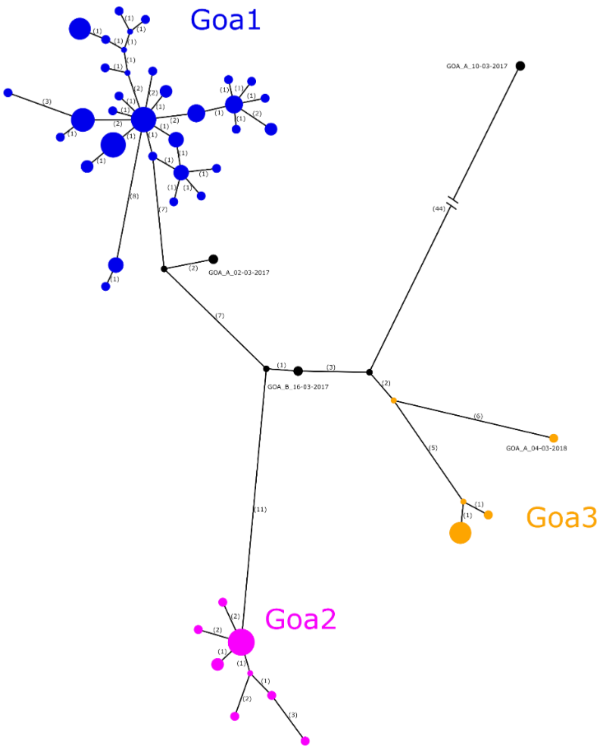

Supplementary Figure 16. Rabies virus glycoprotein haploid network.
Haplotype network analysis of 97 Goa rabies virus glycoprotein gene sequences using the Median joining network approach. Numbers in parentheses represent the number of changes between two nodes. The circle size represents the number of identical sequences. Line connecting translocation case GOA_A_10-03-2017 is shortened for viewing convenience (as indicated by the breaks). Sample ID is given for samples that were not assigned to one of the three groups and for sample GOA_A_04-03-2018, due to divergence from other Group 3 samples. Three rabies virus sequences did not fit into any group using the cut-off criteria described in the Methods. Sample Goa_A_02-03-2017 displayed a high identity to Group 1 sequences but was more diverged. Goa_B_16-03-2017 displayed similar nucleotide identity to glycoprotein sequences in Groups 1, 2, and 3. Goa_A_10-03-2017 displayed <97% identity to all other samples and was identified as an importation case into Goa.

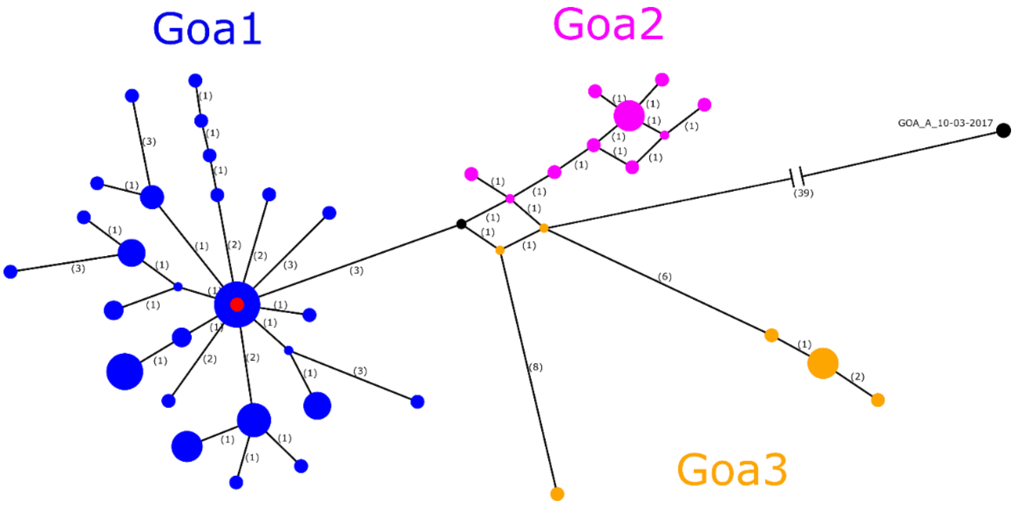

Supplementary Figure 17. Rabies virus nucleoprotein haploid network.
Haplotype network analysis of 80 Goa rabies virus nucleoprotein gene sequences using the Median joining network approach. Numbers in parentheses represent the number of changes between two nodes. The circle size represents the number of identical sequences. Line connecting translocation case GOA_A_10-03-2017 is shortened for viewing convenience (as indicated by the breaks). Location of discrepant sample GOA_B_16-03-2017 is shown in red.

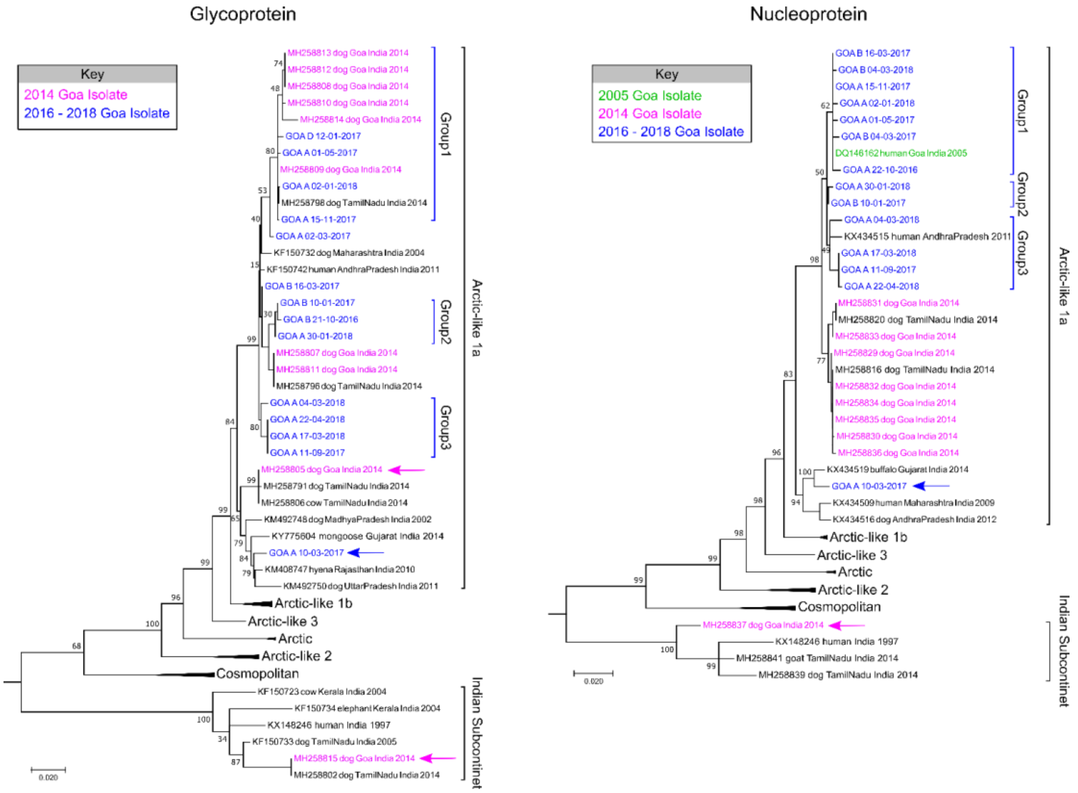

Supplementary Figure 18. Phylogenetic analysis of Goa rabies virus gene sequences in relation to India reference sequences.

Phylogenetic analysis of rabies virus glycoprotein (left) and nucleoprotein (right) gene sequences from Goa, India and reference sequences from India. Phylogenetic tree estimated by maximum likelihood method (GTR+G+I). Numbers on branch points represent bootstrap support based on 1000 replicates. Sample IDs from Goa rabies cases are colored by study. Blue arrows highlight an importation case into a neighboring region to Goa from northern India. Magenta arrows highlight two divergent Goa rabies virus case from 2014. A representative subset of the 97 sequences from 2016 – 2018 were included in the analysis for ease of view. Rabies virus lineage designation was based on Troupin et al. (2016)[15]. Group1, Group2, and Group3 designations correspond to 2016 – 2018 Goa sequence groupings (Fig. 3). Reference sequences are labelled with GenBank accession number, host, location and year, if available. Sequences used for collapsed lineages can be found in the Methods. Scale bar indicates changes per site.

Partial glycoprotein sequences from a 2014 study reporting ten samples from Goa[16] were compared with the newly generated sequences. The availability of only partial sequences resulted in low confidence values for some branches. However, eight 2014 samples clustered with the 2016 – 2018 sequences (Groups 1, 2 and 3) from the current study. Two 2014 samples did not group with 2016 – 2018 sequences: one was on a sister branch within the Arctic-like 1a lineage, but the other grouped in the Indian Subcontinent rabies virus lineage, which is highly diverged. Eight corresponding nucleoprotein gene sequences from 2014 and a partial sequence from a 2005 Goa sample[17] also grouped with the 2016 – 2018 sequences, while one 2014 sample fell in the Indian subcontinent lineage.

Comparison of the 97 glycoprotein coding sequences from Goa with publicly available reference sequences revealed all sequences fell into the Arctic-like 1a lineage of rabies virus, and clustered with sequences from Southern India. The newly generated sequences were most similar to partial G gene sequences collected in Goa in 2014; however, the availability of only partial sequences limited the analysis and resulted in low confidence values in phylogenetic groupings. All 2016 – 2018 Goa samples clustered with one subset of Goa sequences from 2014 (MH258807, MH258809, MH258810, MH258811, MH258813, and MH258814, Arctic 1a) and not with MH258805 (Arctic-like 1a) or MH258815 (Indian Subcontinent). The 2014 Goa sequences clustered with Group 1 and 2 sequences generated as part of this study, while a sample of a human rabies case from Andhra Pradesh in 2011 clustered with group 3 sequences; however, confidence in these groupings was very low. Nucleoprotein comparison with publicly available sequences produced concordant findings; the 2016 – 2018 sequences formed a branch with 2014 Goa N gene sequences and sample from a human rabies case, infected in Goa in 2005 (KX434515). Similar to the G gene analysis, the 2016 – 2018 N gene sequences clustered with the 2014 Goa sequences in the Arctic 1a lineage, and no sequences clustered with the Indian subcontinent sample. The 2014 Goa N gene sequence for the more divergent Arctic-like 1a sample (corresponding to G gene sequence MH258805) was only 90 bp and was not included in the analysis.

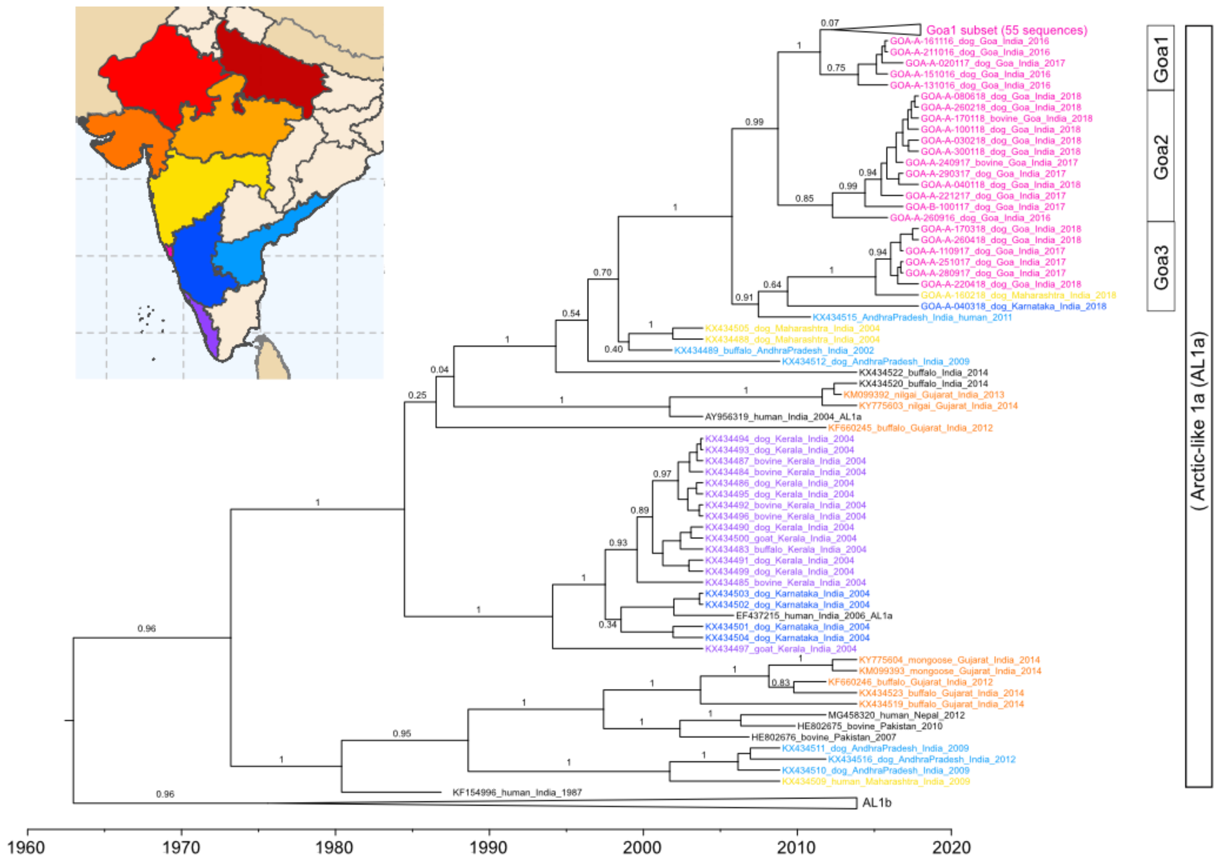

Supplemental Figure 19. Time-scaled phylogeny of nucleoprotein gene sequences in relation to India reference sequences.

Time-scaled phylogeny of complete nucleoprotein gene sequences of Goa samples, Karnataka sample, and Maharashtra sample generated in this study with representative reference sequences from India belonging to the Arctic-like 1a rabies virus lineage. Samples from Cosmopolitan and Indian Subcontinent lineages were excluded from this analysis. Scale at the bottom indicates year. Sample IDs are colored based on the state of sample collection, according to the coloration on the map. Bars to the right indicate members of Goa1, Goa2, and Goa3 groups. Numbers at the branches indicate posterior support values. Estimated dates for the most recent common ancestor of all Goa, Karnataka, and Maharashtra samples generated in this study was 2005 (2002.6 – 2009.0). Ages of Goa1, Goa2, Goa3, and Goa3a were 2011.5 (2009.5 – 2013.9), 2011.7 (2007.8 – 2015.3), 2008.7 (2004.6 – 2012.6), and 2015.0 (2012.7 – 2016.6), respectively. AL1b: Arctic-like 1b. The observed slight differences in estimated ages from time-scaled phylogeny of partial glycoprotein gene sequences likely reflects the different sequences used in the two analyses, as no nucleoprotein sequences were produced for 16 Goa samples in this study and only nucleoprotein or glycoprotein gene sequences were available for several publicly available references.

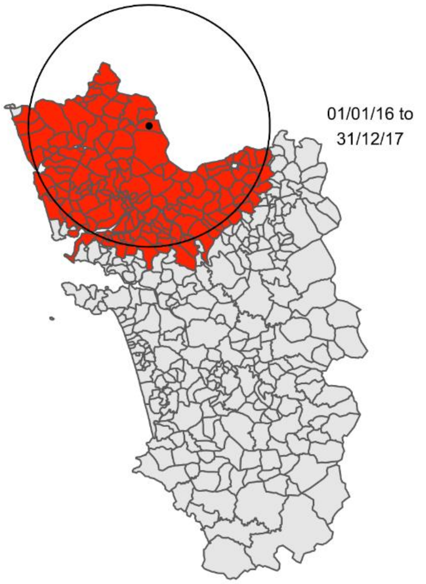

Supplementary Figure 20. Spatio-temporal statistical analysis.
Map of Goa village panchayats showing the statistically significant canine rabies cluster as determined from space-time statistical analysis of canine rabies case data from 2014 to 2019. The statistically significant cluster ($p < 0.05$) is defined by the black circle with panchayat areas within the cluster coloured red. The dates during which this cluster was considered significant are shown in the top-right hand corner of the figure.

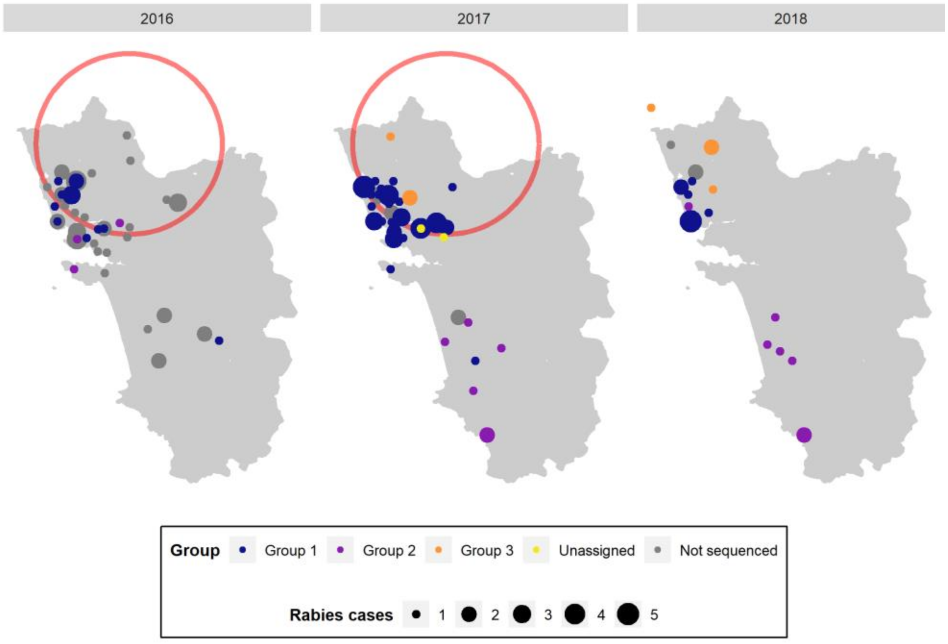

Supplementary Figure 21. Phylogenetic distribution and statistically significant spatio-temporal clustering of rabies virus cases by year.
Map of Goa showing geographic distribution of positive rabies cases, phylogenetic groupings and statistically significant spatio-temporal cluster (red circle) from 2016 to 2018. The location of rabies cases not sequenced during this period are also included.

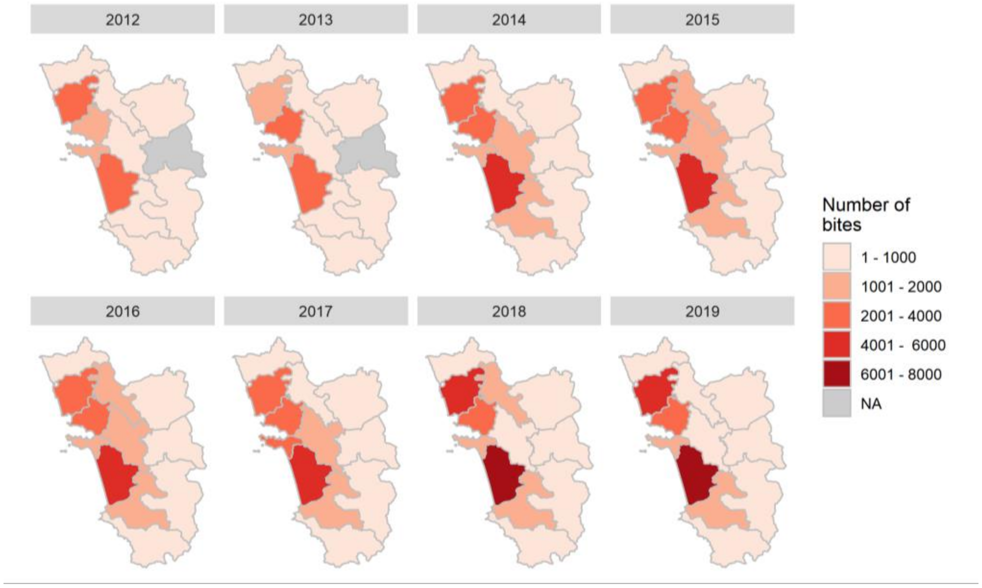

Supplementary Figure 22. Human dog bites reported by taluka and year.
Choropleth maps of Goa showing the number of human dog bites reported to medical facilities by taluka from 2012 to 2019.

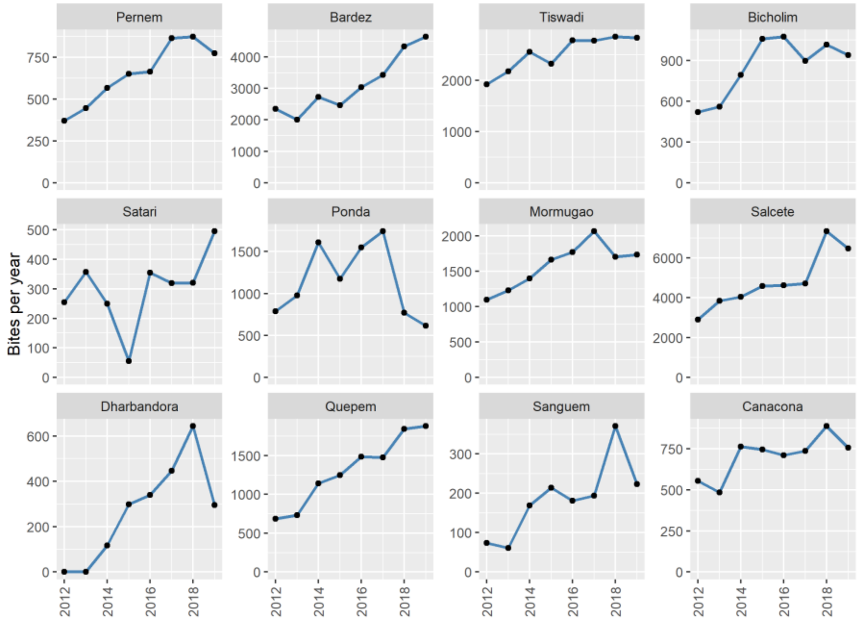

Supplementary Figure 23. Human dog bite by taluka and year.
Year-wise total number of human dog bites reported to medical facilities by taluka from 2012 to 2019.

Supplementary Tables

Supplementary Table 1. Intervention outputs for dog vaccination and education activities.

Year	Dog vaccinations						Education activities			
	Total dogs vaccinated	Proportion villages vaccinated (%)	Number of post-vacc surveys	Proportion villages surveyed (%)	Estimated total dog vaccination coverage (%)	Estimated roaming dog vaccination coverage (%)	No. children taught	No. teachers taught	Community reach (people educated)	No. schools
**2013**	5,092	4.3								
**2014**	22,059	7.4	168	20.7		47.5 (45 - 50.1)	72,744	3,024	1,122	
**2015**	56,954	57.4	360	40.1		37.7 (35.3 - 40.1)	40,070	2,589	25,205	
**2016**	51,302	43.4	323	43.9	71.8 (70.3 - 73.3)	60.1 (58.2 - 62.1)	62,782	3,054	23,012	
**2017**	97,277	86.5	847	87	71.7 (70.9 - 72.6)	53.1 (51.7 - 54.5)	172,728	7,162	22,293	1,368
**2018**	97,248	96.2	512	53.3	68.7 (67.5 – 70.0)	47.3 (45.5 – 49.0)	171,097	7,339	52,503	1,427
**2019**	96,187	81.9	125	12.8	77.5(75.3 – 79.7)	59.5 (56.0 – 63.0)	174,850	8,083	30,944	1,456

Supplementary Table 2. Indicators for human and canine rabies incidence.

The calculation of mean positive canine rabies cases per month was based on months in which surveillance was active.

Year	Estimated human population[18]	Human surveillance				Canine surveillance								
		Human deaths	Human deaths per 100,000 capita	Total bites	Est bites per 100,000 capita	Total calls	Mean calls/ week	Proportion calls rabies suspect (%)	Total in-person investigated	Total tested	Canine positive cases	Tested proportion positive (%)	Total months active	Mean positive/ month
**2012**	1,466,020	13	0.89	11,515	785									
**2013**	1,473,384	5	0.34	12,857	873									
**2014**	1,480,636	17	1.15	16,136	1,090				111	94	74	78.7	7	10.6
**2015**	1,487,779	5	0.34	16,478	1,108				46	45	39	86.7	4	9.8
**2016**	1,494,812	1	0.07	18,585	1,243				84	78	64	82.1	12	5.3
**2017**	1,501,737	2	0.13	19,655	1,309	628	57.1	3.2	164	132	81	61.4	12	6.8
**2018**	1,508,556	0	0.00	22,955	1,522	2561	50.2	2.3	82	73	29	39.7	12	2.4
**2019**	1,515,268	0	0.00	21,662	1,430	4183	78.9	1.2	133	130	9	6.9	12	0.8

Supplementary Table 3. Table of granted amounts allocated and received from the Government of Goa for implementation of rabies control activities during the study period (2013 – 2019).

Block	Grant ID	Grant period	Instalment allocated (INR)	Total received (INR)	Months covered	Amount per month (INR)
Grant 1	**g1_1**	Sep 15 - Feb 16	2,700,000	2,783,000	6	463,833
	**g1_2**	Mar 16 - Aug 16	2,700,000	2,028,240	6	338,040
Grant 2	**g2_1**	Sep 16 - Feb 17	2,700,000	1,902,739	6	317,123
	**g2_2**	Mar 17 - Aug 17	2,700,000	2,262,200	6	377,033
Grant 3	**g3_1**	Sep 17 - Feb 18	2,700,000	3,075,800	6	512,633
	**g3_2**	Mar 18 - Aug 18	2,700,000	2,553,800	6	425,633
**Grant 4**	**g4_1**	Sept 18 - Aug 19	9,198,600	8,688,600	12	724,050
**Grant 5**	**g5_1**	Sept 19 - Aug 20	9,198,600	9,198,600	12	766,550

Supplementary Table 4. Calculation of annual (Jan - Dec) Goa Government grant allocation.

The funding allocation from the Government of Goa grant spanned different timeframes and so needed to standardise to the Jan – Dec cycle for inclusion in other calculations. This table provides the calculations for grant expenditure by month to calculate annual (Jan – Dec) granted expenditure during the study period.

Year	Grant ID	Grant monthly amount (INR)	Months covered	Number of months	Year instalment amount (INR)	Year total (INR)
2015	g1_1	463,833	Sep 15 - Dec 15	4	1,855,333	1,855,333
2016	g1_1	463,833	Jan 16 - Feb 16	2	927,667	4,224,399
2016	g1_2	338,040	Mar 16 - Aug 16	6	2,028,240	
2016	g2_1	317,123	Sep 16 - Dec 16	4	1,268,493	
2017	g2_1	317,123	Jan 17 - Feb 17	2	634,246	4,946,980
2017	g2_2	377,033	Mar 17 - Aug 17	6	2,262,200	
2017	g3_1	512,633	Sep 17 - Dec 17	4	2,050,533	
2018	g3_1	512,633	Jan 18 - Feb 18	2	1,025,267	6,475,267
2018	g3_2	425,633	Mar 18 - Aug 18	6	2,553,800	
2018	g4_1	724,050	Sep 18 - Dec 18	4	2,896,200	
2019	g4_1	724,050	Jan 19 - Aug 19	8	5,792,400	8,858,600
2019	g5_1	766,550	Sep 19 - Dec 19	4	3,066,200	

Supplementary Table 5. Table of campaign expenditure and estimated vaccine value for the calculation of annual estimated campaign value and mean cost per dog vaccinated.

		2015	2016	2017	2018	2019
**US exchange rate***		64.15	67.20	65.07	68.30	70.40
**Vaccine contribution**	Total dog vaccinations	56,954	51,302	97,277	97,248	96,187
	Estimated value per vaccine (USD)	0.53	0.53	0.53	0.53	0.53
	Estimated value per vaccine (INR)	34	36	34	36	37
	**Estimated total vaccine value (INR)**	**1,936,381**	**1,827,095**	**3,354,844**	**3,520,462**	**3,588,978**
**FULL PROGRAMME EXPENDITURE** (dog vaccination / education / surveillance)**						
**Total Expenditure****	Donated fund domestic expenditure (INR)***	7,067,842	8,725,573	18,934,887	15,346,996	12,471,346
	Goa Government funding expenditure (INR)	1,855,333	4,224,399	4,946,980	6,475,267	8,858,600
	**Total Goa expenditure (INR)**	**8,923,175**	**12,949,973**	**23,881,867**	**21,822,263**	**21,329,946**
**Total programme cost (vaccine value + expenditure)**	**Total estimated campaign value (INR)**	**10,859,556**	**14,777,068**	**27,236,711**	**25,342,725**	**24,918,925**
	**Total estimated campaign value (USD)**	**169,286**	**219,906**	**418,570**	**371,031**	**353,957**
**Cost per dog vaccinated (total programme)**	**INR**	**191**	**288**	**280**	**261**	**259**
	**USD**	**2.97**	**4.29**	**4.30**	**3.82**	**3.68**
**DOG VACCINATION CAMPAIGN EXPENDITURE**						
**Dog vaccination Expenditure**	Donated fund domestic expenditure (INR)				13,833,503	9,952,588
	Goa Government funding expenditure (INR)				6,475,267	8,858,600
	**Goa dog vaccination expenditure (INR)**				**20,308,770**	**18,811,188**
**Dog vaccination campaign cost (vaccine value + expenditure)**	**Total estimated campaign value (INR)**				**23,829,232**	**22,400,167**
	**Total estimated campaign value (USD)**				**348,873**	**318,180**
**Cost per dog vaccinated (dog vaccination campaign)**	**INR**				**245**	**233**
	**USD**				**3.59**	**3.31**

* Mean annual US dollar exchange rates were calculated from the International Monetary Fund country database records: https://www.imf.org/external/np/fin/ert/GUI/Pages/CountryDataBase.aspx

** Expenditure values were only available throughout 2015 – 2019 at the campaign level and therefore include the cost of dog vaccination, education and rabies surveillance activities.

*** Donated expenditure constitutes only campaign implementation expenditure in Goa. International expenditure from Mission Rabies in project administration, data analysis and smartphone technology development were not included as these would not be associated with routine vaccination campaign implementation.

Supplementary Table 6. Multivariable mixed effects logistic regression model predicting a taluka having at least one confirmed dog rabies case in a particular month. Explanatory variables investigated included free roaming dog population, free roaming dog population density, estimated monthly vaccination coverage, estimated 12-month rolling mean coverage, season and whether the taluka borders unvaccinated dog regions (Supplementary Data 1). The data were randomly split into a training and testing dataset using a 70:30 ratio using R package caret. Univariable analysis was used and any variable with a p-value of <0.15 was considered for the final model. To investigate whether numerical variables had a linear relationship with the log-odds of the outcome, these were split into quartiles and univariable models were visualized to assess the relationship. Manual forward variable selection was conducted and the final model was chosen based on the lowest Akaike information criterion (AIC). The final model was validated, testing its ability to predict the outcome in the test dataset by estimating the area under the curve using R package ROCR. The p value for vaccination coverage was 1.11e-06.

Variable	estimate	conf.low	conf.high	std.error	p.value
Dog population density (centered)	8.448	2.343	30.465	0.654	0.001
Vaccination coverage (12 month rolling average - centered)	0.176	0.088	0.354	0.356	<0.001
Season: Rainy	2.339	0.929	5.891	0.471	0.071
Season: Summer	2.013	0.74	5.473	0.51	0.171
Season: Winter	3.423	1.311	8.939	0.49	0.012

Supplementary Table 7. Average nucleotide identity of rabies virus glycoprotein coding region.

Range (minimum – maximum) pairwise nucleotide identities are shown below means in parenthesis. Shading indicates range included values ≥99% identity.

	Group 1	Group 2	Group 3	GOA_B_16-03-2017	GOA_A_02-03-2017	GOA_A_10-03-2017
Group 1	**99.699** (99.111 - 100)					
Group 2	**98.301** (98.030 - 98.603)	**99.867** (99.524 - 100)				
Group 3	**98.279** (98.094 - 98.603)	**98.490** (98.317 - 98.540)	**99.782** (99.238 - 100)			
GOA_B_16-03-2017	**98.962** (98.793 - 99.175)	**99.189** (99.016 - 99.238)	**99.302** (99.302 - 99.302)	**100**		
GOA_A_02-03-2017	**99.187** (98.889 - 99.397)	**98.672** (98.517 - 98.792)	**98.651** (98.635 - 98.762)	**99.333**	**100**	
GOA_A_10-03-2017	**96.016** (95.810 - 96.190)	**96.204** (96.032 - 96.254)	**96.714** (96.698 - 96.825)	**97.016**	**96.349**	**100**

Additional supplementary files

Supplementary Data 1 - Logistic regression model data set

Supplementary Data 2 - Phylogenetic sample data set

Supplementary Data 3 – RabiesEcon model

Supplementary Software 1 – R code for Logistical Regression analysis

Supplementary Software 2 – R code for Spatio-temporal analysis

Supplementary References

1. Wallace, R. M. *et al.* Establishment of a canine rabies burden in Haiti through the implementation of a novel surveillance program. *PLoS Negl Trop Dis* **9**, e0004245 (2015).
2. Animal Welfare Board of India. *Criteria and standards of euthanasia of animals*. 41 (2013).
3. Yale, G. *et al.* Evaluation of an immunochromatographic assay as a canine rabies surveillance tool in Goa, India. *Viruses* **11**, 1–10 (2019).
4. Tenzin, T., Ahmed, R., Debnath, N. C., Ahmed, G. & Yamage, M. Free-Roaming Dog Population Estimation and Status of the Dog Population Management and Rabies Control Program in Dhaka City, Bangladesh. *PLoS Negl. Trop. Dis.* **9**, e0003784 (2015).
5. Gigante, C. M. *et al.* Portable Rabies Virus Sequencing in Canine Rabies Endemic Countries Using the Oxford Nanopore MinION. *Viruses* **12**, 1255 (2020).
6. Katoh, K. & Standley, D. M. MAFFT multiple sequence alignment software version 7: Improvements in performance and usability. *Mol. Biol. Evol.* **30**, 772–780 (2013).
7. Kumar, S., Stecher, G. & Tamura, K. MEGA7: Molecular Evolutionary Genetics Analysis Version 7.0 for Bigger Datasets. *Mol. Biol. Evol.* **33**, 1870–1874 (2016).
8. Leigh, J. W. & Bryant, D. POPART: Full-feature software for haplotype network construction. *Methods Ecol. Evol.* **6**, 1110–1116 (2015).
9. Bandelt, H.-J., Foster, P. & Rohl, A. Median-joining networks for inferring intraspecific phylogenies. *Mol. Biol. Evol.* **16**, 37–48 (1999).
10. Hijmans, R. J. raster: Geographic Data Analysis and Modeling. (2020).
11. Wickham, H. *Ggplot2: Elegant Graphics for Data Analysis*. (2016).
12. Dunnington, D. & Thorne, B. ggspatial: Spatial Data Framework for ggplot2. (2018).
13. GADM. GADM maps and data. https://gadm.org/ (2020).
14. Kassambara, A. ggscatter: Scatter plot. (2020).
15. Troupin, C. *et al.* Large-Scale Phylogenomic Analysis Reveals the Complex Evolutionary History of Rabies Virus in Multiple Carnivore Hosts. *PLoS Pathog.* **12**, e1006041 (2016).
16. Deventhiran, J. *et al.* Continued circulation, recombination and evolution of the ancient subcontinent lineage despite predominance of the recent arctic-like lineage rabies viruses (RABV) in India. *bioRxiv (preprint)* 398180 (2018) doi:10.1101/398180.
17. Solomon, T. *et al.* Paralytic rabies after a two week holiday in India. *Br. Med. J.* **331**, 501–503 (2005).
18. Government of India, Ministry of Home Affairs & Office of the Registrar General & Census Commissioner. *Census of India*. https://www.census2011.co.in/ (2011).
