# Supplementary material for: Elimination of human rabies in Goa, India through an integrated One Health approach
Source: Nat Commun. 2022 May 19;13:2788. doi: 10.1038/s41467-022-30371-y (PMC9120018; doi:10.1038/s41467-022-30371-y)
Supplement: Supplementary file 2 — Description of Additional Supplementary Files [file 41467_2022_30371_MOESM2_ESM.pdf]

Description of Additional Supplementary Files

File Name: Supplementary Data 1
Description: Logistic regression model data set

File Name: Supplementary Data 2
Description: Phylogenetic sample data set

File Name: Supplementary Data 3
Description: RabiesEcon model

File Name: Supplementary Software 1
Description: R code for Logistical Regression analysis

File Name: Supplementary Software 2
Description: R code for Spatio-temporal analysis
