# Supplementary material for: Elimination of human rabies in Goa, India through an integrated One Health approach
Source: Nat Commun. 2022 May 19;13:2788. doi: 10.1038/s41467-022-30371-y (PMC9120018; doi:10.1038/s41467-022-30371-y)
Supplement: Supplementary file 8 — Reporting Summary [file 41467_2022_30371_MOESM8_ESM.pdf]

nature portfolio

Corresponding author(s): Andrew D Gibson

Last updated by author(s): Mar 16, 2022

Reporting Summary

Nature Portfolio wishes to improve the reproducibility of the work that we publish. This form provides structure for consistency and transparency in reporting. For further information on Nature Portfolio policies, see our Editorial Policies and the Editorial Policy Checklist.

Statistics

For all statistical analyses, confirm that the following items are present in the figure legend, table legend, main text, or Methods section.

n/a	Confirmed	
☐	☒	The exact sample size (*n*) for each experimental group/condition, given as a discrete number and unit of measurement
☐	☒	A statement on whether measurements were taken from distinct samples or whether the same sample was measured repeatedly
☐	☒	The statistical test(s) used AND whether they are one- or two-sided<br>*Only common tests should be described solely by name; describe more complex techniques in the Methods section.*
☐	☒	A description of all covariates tested
☐	☒	A description of any assumptions or corrections, such as tests of normality and adjustment for multiple comparisons
☐	☒	A full description of the statistical parameters including central tendency (e.g. means) or other basic estimates (e.g. regression coefficient) AND variation (e.g. standard deviation) or associated estimates of uncertainty (e.g. confidence intervals)
☐	☒	For null hypothesis testing, the test statistic (e.g. *F*, *t*, *r*) with confidence intervals, effect sizes, degrees of freedom and *P* value noted<br>*Give P values as exact values whenever suitable.*
☒	☐	For Bayesian analysis, information on the choice of priors and Markov chain Monte Carlo settings
☒	☐	For hierarchical and complex designs, identification of the appropriate level for tests and full reporting of outcomes
☐	☒	Estimates of effect sizes (e.g. Cohen's *d*, Pearson's *r*), indicating how they were calculated

Our web collection on statistics for biologists contains articles on many of the points above.

Software and code

Policy information about availability of computer code

Data collection	Data were collected from dog vaccination, rabies surveillance and community education field activities using the WVS App (Android versions 1.0 - 6.6) in accordance with the MOU between Mission Rabies and the Government of Goa to implement the project. Records were verified with paper-based project records.<br>Data on human rabies deaths and human dog bites were gathered from the Government of Goa Directorate of Health Services data reports.
Data analysis	Data were analyzed using R statistical software version 3.6.2 and 4.0.2.<br>Cluster analyses was performed using SaTScan™ v9.6 software through R package rsatscan.<br>Cost-effectiveness analysis was conducted using the MS Excel based model, RabiesEcon published by Kunkel et al (2022), with modification described in the methods.<br>Phylogenetic analysis was performed using Mega7 and BEAST v1.10.4 and phylogeny trees were generated using FigTree v1.4.4.

For manuscripts utilizing custom algorithms or software that are central to the research but not yet described in published literature, software must be made available to editors and reviewers. We strongly encourage code deposition in a community repository (e.g. GitHub). See the Nature Portfolio guidelines for submitting code & software for further information.

Data

Policy information about availability of data

All manuscripts must include a data availability statement. This statement should provide the following information, where applicable:

- Accession codes, unique identifiers, or web links for publicly available datasets
- A description of any restrictions on data availability
- For clinical datasets or third party data, please ensure that the statement adheres to our policy

Data supporting the findings of this work are available within the paper and its Supplementary Information files.
All sequences generated as part of this study are deposited in GenBank (accession codes: MW054945-MW055041).
GenBank accession codes for reference sequences used in the phylogenetic analysis were as follows:
JQ685901, JQ685945, JQ944707, KX148105, JQ944708, JQ944706, JQ944705, KX148225, HE801587, JX987739, KF150744, MK760761, KX148226, HE802676, KC171645, KC171644, KC171643, KM272192, GU937029, KX148228.

Field-specific reporting

Please select the one below that is the best fit for your research. If you are not sure, read the appropriate sections before making your selection.

☒ Life sciences ☐ Behavioural & social sciences ☐ Ecological, evolutionary & environmental sciences

For a reference copy of the document with all sections, see nature.com/documents/nr-reporting-summary-flat.pdf

Life sciences study design

All studies must disclose on these points even when the disclosure is negative.

Sample size	The vaccination approach aimed to access as high a proportion of the population as possible. A total of 426,119 dog rabies vaccination events were conducted during the study period. Postmortem sampling and rabies diagnostic testing was performed on 552 dogs with suspect rabies as part of the Goa State rabies surveillance activities coordinated by the Department for Animal Husbandry and Veterinary Services and in accordance with The Prevention of Cruelty to Animals Act, 1960. Attempts were made to sample all dogs reported to have died in Goa following signs consistent with rabies (aggression, hypersalivation, neurological signs, sudden death).
Data exclusions	No data were excluded from the study.
Replication	The study demonstrates strategies for effective human and canine rabies elimination at the sub-district administrative region (taluka), which is a standard administrative unit in India. Our logistical regression analysis assessed the relationship between canine rabies and dog vaccination coverage across twelve taluka regions. The odds of dog rabies decreased as rolling mean 12-month vaccination coverage increased, a pattern that was seen across 10 of the 12 talukas, with identified causes of the two anomalies included in the discussion. As such the outcome of human and canine rabies elimination through the described methods would be repeatable under similar epidemiological settings in India.
Randomization	As this was a state-wide initiative all regions of the state were included in the study. It was not considered appropriate or feasible to perform a randomized cohort study on the implementation aspect of the study from a financial, political or ethical standpoint. We have accounted for covariates where appropriate, e.g. with the use of multivariable logistic regression.<br>Samples for phylogenetic analysis were randomly selected from the bank of positive rabid dog brains spanning 2016 - 2018 using a random number generator.
Blinding	As all areas of the state were included in the rabies control program, it was not possible to blind investigators during data collection or analysis.<br>Individuals performing laboratory diagnosis were blinded to the initial Lateral Flow Test outcome.<br>Individuals performing post-vaccination surveys were blinded from the areas that had been vaccinated.

Reporting for specific materials, systems and methods

We require information from authors about some types of materials, experimental systems and methods used in many studies. Here, indicate whether each material, system or method listed is relevant to your study. If you are not sure if a list item applies to your research, read the appropriate section before selecting a response.

Materials & experimental systems

n/a	Involved in the study	
☒	☐	Antibodies
☒	☐	Eukaryotic cell lines
☒	☐	Palaeontology and archaeology
☐	☒	Animals and other organisms
☒	☐	Human research participants
☒	☐	Clinical data
☒	☐	Dual use research of concern

Methods

n/a	Involved in the study	
☒	☐	ChIP-seq
☒	☐	Flow cytometry
☒	☐	MRI-based neuroimaging

Animals and other organisms

Policy information about studies involving animals; ARRIVE guidelines recommended for reporting animal research

Laboratory animals	Laboratory animals were not involved in any part of this study.
Wild animals	Over 400,000 events of the vaccination of owned and unowned domestic dogs were recorded as part of the study. Dogs of any age and sex were vaccinated in accordance with government and WHO guidance on mass dog vaccination for rabies control. The study activities were part of the state public health intervention to control the spread of rabies. Dogs were handled humanely or using butterfly nets by trained staff in accordance with state and national guidelines. Dogs were vaccinated by subcutaneous or intramuscular injection, marked with a non-toxic temporary marker paint and immediately released at the same location. For owned dogs, consent was required before conducting vaccination.
Field-collected samples	Postmortem brain samples were collected from suspect rabid animals in Goa during the study period. Samples were taken as soon after death as possible at the Disease Investigation Unit in Tonca, Panjim. Samples were stored at 4 degrees Celsius until processing for rabies diagnosis using direct fluorescent antibody testing within days. A subset were stored a -80C for subsequent quality-control and viral gene sequencing. Samples continue to be stored by the Government of Goa as an ongoing archive of rabies virus for subsequent analysis. When samples are no longer required, they are incinerated as hazardous biological waste.
Ethics oversight	The Government of Goa Department for Animal Husbandry oversaw the project protocols and methods for mass dog vaccination and rabies surveillance. The Goa Veterinary Association also had oversight of veterinary protocols on the project.<br>Veterinary ethical review was also provided by the University of Edinburgh Veterinary Ethical Review Committee, with ethical approval granted on 11th Dec 2014 (107 14: Investigation of efficacy of rabies vaccination in India) and 28th July 2015 (64/15: Investigation of rabies vaccination approaches).

Note that full information on the approval of the study protocol must also be provided in the manuscript.
